# Transcriptional Dysregulation Study Reveals a Core Network Involving the Progression of Alzheimer's Disease

**DOI:** 10.3389/fnagi.2019.00101

**Published:** 2019-05-07

**Authors:** Guofeng Meng, Hongkang Mei

**Affiliations:** ^1^Institute of Interdisciplinary Integrative Biomedical Research, Shanghai University of Traditional Chinese Medicine, Shanghai, China; ^2^Computational and Modeling Science, PTS China, GSK R&D, Shanghai, China

**Keywords:** Alzheimer's disease, co-expression, dysregulation, network, divergence, transcriptome

## Abstract

**Background:** The pathogenesis of Alzheimer's disease is associated with dysregulation at different levels from transcriptome to cellular functioning. Such complexity necessitates investigations of disease etiology to be carried out considering multiple aspects of the disease and the use of independent strategies. The established works more emphasized on the structural organization of gene regulatory network while neglecting the internal regulation changes.

**Methods:** Applying a strategy different from popularly used co-expression network analysis, this study investigated the transcriptional dysregulations during the transition from normal to disease states.

**Results:** Ninety- seven genes were predicted as dysregulated genes, which were also associated with clinical outcomes of Alzheimer's disease. Both the co-expression and differential co-expression analysis suggested these genes to be interconnected as a core network and that their regulations were strengthened during the transition to disease states. Functional studies suggested the dysregulated genes to be associated with aging and synaptic function. Further, we checked the conservation of the gene co-expression and found that human and mouse brain might have divergent transcriptional co-regulation even when they had conserved gene expression profiles.

**Conclusion:** Overall, our study reveals a core network of transcriptional dysregulation associated with the progression of Alzheimer's disease by affecting the aging and synaptic functions related genes; the gene regulation is not conserved in the human and mouse brains.

## 1. Introduction

Alzheimer's disease (AD) is a neurodegenerative disorder most prevalent in people over the age of 65 years (Lashuel et al., 2002; Goedert and Spillantini, 2006; Prince et al., 2013). As the population ages, AD will impact more people and place an increasing economic burden on society (Cummings et al., 2014; Alzheimer's, 2015). There is still no effective treatment that prevents or slows the disease progression. A significant challenge is the poor understanding of the etiology of this disease (Krstic and Knuesel, 2013; Karch and Goate, 2015; Kumar et al., 2015). The progression of AD is associated with the dysregulation of many genes at different regulatory levels from transcriptome to neuronal function (Minati et al., 2009; Huang and Mucke, 2012). To study such a complex multifactorial disease, integrated and large-scale data are necessary to catch the diverse regulatory interactions (Zhang et al., 2013; Norton et al., 2014). The availability of high-throughput transcriptomic sequencing data and clinical annotation in the Accelerating Medicines Partnership-Alzheimer's Disease (AMP-AD) program (provides an opportunity to study the altered transcriptional regulation during the progression of AD (Myers et al., 2007; Webster et al., 2009; Zou et al., 2012; Zhang et al., 2013).

Co-expression network analysis is useful to infer causal mechanisms for complex diseases (Stuart et al., 2003; Miller et al., 2010; Oldham et al., 2012). It is based on the assumption that co-expressed genes are usually regulated by the same transcriptional regulators, pathways or protein complexes and that the co-regulated genes can be revealed by analysis of the topological structure of co-expression networks (Horvath et al., 2006; Villa-Vialaneix et al., 2013; Zhang et al., 2015). The co-regulated clusters in the network provide the chance to track the affected pathways or biological processes in diseases (Langfelder and Horvath, 2007). One of the most popular strategies is to find the topological structural changes of the co-expression network under different disease states where these changes indicate regulatory dysregulation in the disease (Miller et al., 2010; Narayanan et al., 2014). Another way is to associate the subnetwork expression to disease progresses or clinical traits, which can uncover the regulatory components involved in the dysregulation of diseases (McKinney et al., 2015).

Application of co-expression network analysis to AD data has revealed many AD-associated genes and pathways (Zhang et al., 2013). However, the nature of such analyses may bias toward the genes with higher connectivity in a co-expression network. The complexity of AD progression leads us to extend co-expression analysis to a more detailed investigation of co-expressed genes, especially for the genes with relatively low connectivity, during the disease progression. To understand the etiology of AD, the changes of regulation are supposed to be more essential than the regulations themselves. We studied the transcriptional dysregulation by evaluating all the gene pair combinations for their co-expression changes, which can be referred as differential co-expression (DCE) analysis. The genes with altered co-expression are indicated in the progression of AD and their importance can be ranked by the numbers of changed connections.

In this study, we collected the RNA-seq expression data for 1,667 human brain samples from the AMP-AD program. Differential co-expression analysis indicated 87,539 gene pairs to have significant co-expression changes. Among them, 97 genes, including 10 transcription factors, were found to be dysregulated in AD progression. Both the co-expression and differential co-expression analysis suggested these genes to take roles as an interconnected core network. In the transition from normal to disease states, the co-expression is strengthened in this network. Functional studies supported this network to be involved in the etiology of AD by directly or indirectly affecting aging, synaptic function and metabolism related genes. We also evaluated the evolutionary conservation of gene regulations by comparing the co-expression profiles between human and mouse. Dislike the gene expression, the regulation indicated by co-expression is not conserved, including the core network, which may indicate transcriptional regulation divergence between human and mouse.

## 2. Materials and Methods

### 2.1. Data Collection, Processing, and Quality Control

The human expression data were collected from Accelerating MedicinesPartnership-Alzheimer'sDisease (AMP-AD, https://www.synapse.org/#!Synapse:syn2580853/wiki/66722) program compiling with the data access control at http://dx.doi.org/doi.10.7303/syn2580853. The RNA-seq expression data from four projects were used, including (1) ROSMAP; (2) MSBB (3) MayoPilot and (4) MayoBB. Based on the clinical annotation, the samples were grouped as AD and normal samples. In this step, some patients with vague disease status, missing annotation or other disease annotations were filtered out.

For the RNA-seq data from each project, the AD and normal samples were separately processed for quality control. We first performed normalization with the tools of edgeR package (Robinson et al., 2010) for the RNA-seq data with only raw counts. Then, the samples were checked for genomic gene expression similarity. Samples with strong deviation in hierarchical clustering and principal component analysis plots, were treated as outliers and removed. The covariates, such as age, sex, post-mortem interval (PMI), brain regions were evaluated and adjusted by a linear model.

Next, the expression data, including both AD and normal samples, from different projects were treated as different batches and adjusted to remove the batch effects using ComBat (Leek et al., 2012). The adjusted expression data were further normalized with quantile normalization and evaluated by PCA plots to make sure that the selected samples to have consistent expression profiles and have no clear batch effects among the data from different projects. Then, the resulting expression data were divided into two expression profiles for AD and normal samples, respectively.

The mouse and human brain microarray data were collected from GEO database. To minimize the effects of the different microarray platforms, we only selected the data performed with Affymetrix's platforms. Normalized expression data were downloaded and used. For each dataset, we performed quality control to make sure the expression profiles of samples had good expression profile consistency with experimental descriptions introduced in the original paper. The batch effects of the data from different experiments were estimated and removed with ComBat. And then the data were combined together. They were further evaluated for expression profiles consistency and the experiment or sample outliers were removed. Finally, the probes of microarray were mapped to gene symbols. For the genes with multiple probes, we selected the ones with maximum expression values. The gene from human and mouse were mapped based on the gene homologous annotation from Mouse Genome Informatics (MGI) (www.informatics.jax.org).

### 2.2. Differential Co-Expression Analysis

Using the expression values of different samples as elements of expression vectors, the Spearman's correlation of all gene pairs was calculated for AD and normal samples, respectively. To evaluate the robustness of calculated correlations, we performed two simulation studies. In the first evaluation, we randomly selected half of the samples and calculated new correlations. We then checked the mean and variance of correlation values under 100 rounds of simulation. The results helped us to understand the stability of observed correlation values under the different sample selection. In another evaluation, the annotation of samples for each gene was shuffled so that the gene pairs had the wrong sample mapping. The new correlations were calculated under 100 rounds of simulations. This simulation helps us to evaluate the confidence ranges for the observed correlation values.

The correlation differences under disease and normal status were evaluated using the R package DiffCorr (Fukushima, 2013). In this step, the correlation values were transformed with Fisher's transform and z-scores were calculated to indicate the correlation differences. The *p*-values were calculated by fitting to a Gaussian Distribution. After comparing the efficiency, Benjamini and Yekutieli's algorithm in R package (Benjamini and Yekutieli, 2001) was implemented to control the false discovery ratio. At a cutoff of adjusted *p* < 0.01, we select the significantly differentially correlated genes.

### 2.3. Enrichment Analysis

The gene ontology (GO) annotation of gene lists was performed with the GO enrichment analysis tool David (Sherman et al., 2007) under the default setting. The significantly enriched terms for biological process and cellular components were selected at a cutoff of adjusted *p* < 0.05. When multiple gene lists were available, the GO annotation results were visualized in a heatmap to facilitate comparisons.

In this work, we also performed enrichment analysis using an annotated gene list, e.g., text-mining annotated genes. For *k* input genes, the number of genes with annotation is *x*. For *n* whole genomic genes, the number of genes with annotation is *p*. We use Fisher's exact test to evaluate if the observed *x* genes result from random occurrences.

> m=matrix(c(x,k-x,p-x,n-k),ncol=2,byrow=T)
> p=fisher.test(m,alternative="greater")$p.value

### 2.4. Differentially Expressed Genes in Alzheimer's Disease

We performed differential expression analysis to the RNA-seq data collected in above steps from the AMP-AD program. For data from the MSBB project, four brain regions were treated as four independent datasets. Among 7 used datasets, some RNA-seq data had raw counts, e.g., data from MSBB project and we carried out differential expression analysis using edgeR. Other data with normalized expression values were analyzed by log2 transformed *t*-test. For all the datasets, the DEGs were determined at a cutoff of *p* < 0.01. To increase the confidences, the DEGs from different datasets were cross-validated with each other and the ones with clear inconsistency, e.g., the DEG list with weak overlap with other DEG lists were filtered out. Then, the selected DEGs were combined together as the DEGs of AD. The differential expression direction was also checked and determined by using the direction supported by the maximum datasets.

### 2.5. Alzheimer's Disease Related Genes

The AD related genes were determined by selecting the ones with reported association with AD in published works, such as the genetics evidences and expression evidences. In this step, we use the gene-disease annotation for AD from IPA (http://www.ingenuity.com/products/ipa), Metacore (https://portal.genego.com/) and DisGeNet (www.disgenet.org/). We filtered out the low-confidence genes by manually removing the ones with only evidence of expression or those with inconsistency evidences.

### 2.6. Gene Co-expression Network Analysis

Gene co-expression network analysis was to find the gene clusters or modules with good co-expression similarity. As one of well recognized implementation, WGCNA was applied to RNA-seq expression data following the protocol provided by the tool developers https://horvath.genetics.ucla.edu/html/CoexpressionNetwork/Rpackages/WGCNA/ (Langfelder and Horvath, 2008). The RNA-seq data for both AD and normal samples were merged as the input of WGCNA. The power of 6 was determined by pickSoftThreshold function. Then, block-wise network construction were performed by setting the maximum block size to 2,000 and consensus module were detected with a minimum module size of 30. In this step, static height cutoff method was applied to retrieve highly connected modules. All of the expressed genes were clustered into modules, which were labeled with different colors, including gray. In each module, the connectivity and module membership of each module gene were calculated to assess the association and significance of genes in the modules.

## 3. Results

### 3.1. Transcriptional Dysregulation in Alzheimer's Diseases

**Figure 1:**
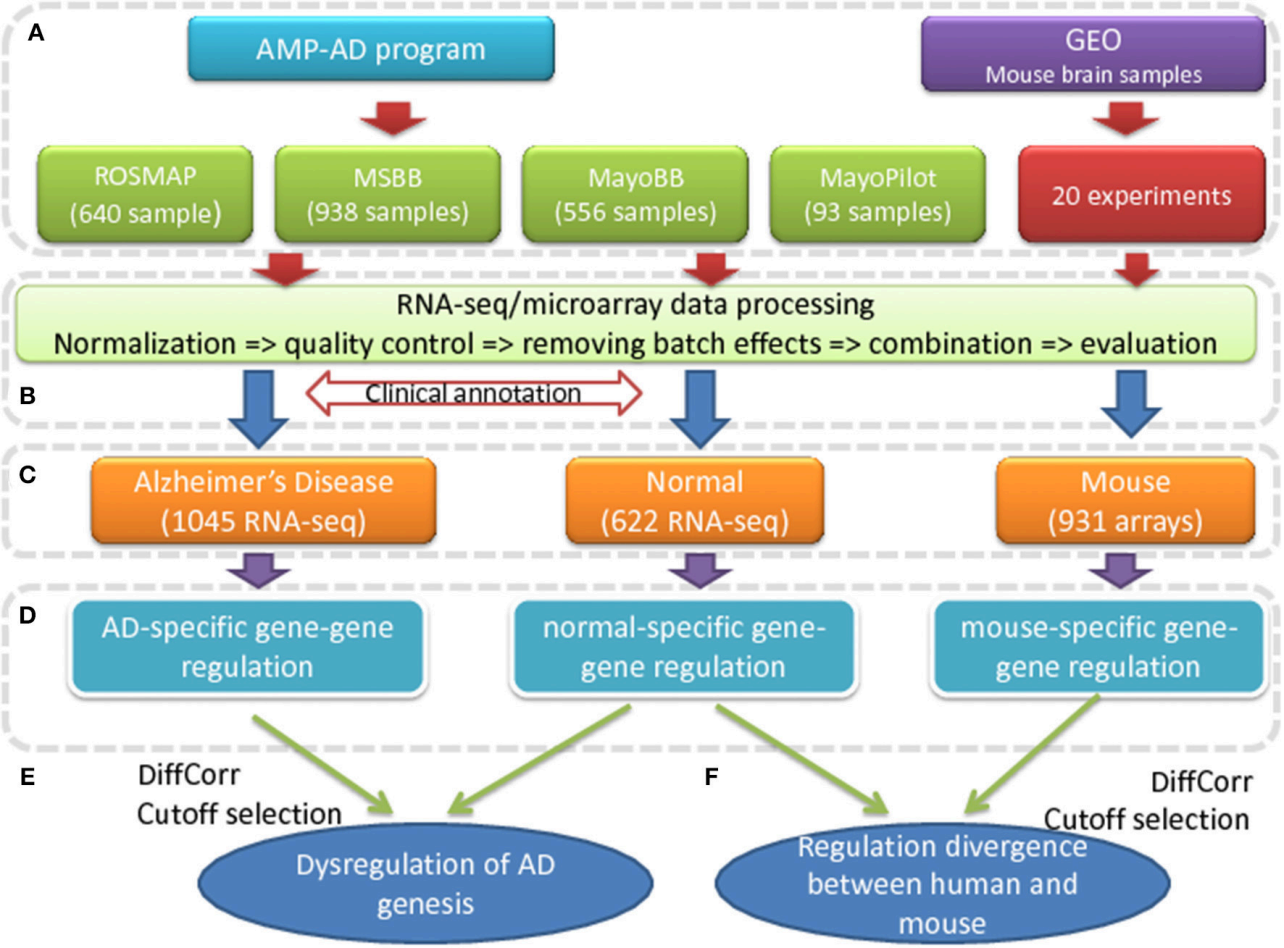
The pipeline to find the transcriptional dysregulation in AD. **(A)** The human RNA-seq expression data are collected from four projects of AMP-AD program; the mouse brain expression data are collected from 20 microarray experiments in GEO database. **(B)** The expression data from different datasets are processed, normalized and combined to describe the expression profiles of AD patients, control subjects and mouse. **(C)** The human brain samples are categorized into AD patients and normal samples based on the clinical annotation. **(D)** All gene pairs are evaluated for gene-gene regulations by measuring co-expression correlations for all the gene pairs. **(E)** The dysregulated genes are predicted by differential co-expression analysis. **(F)** The co-expression conservation of all gene pairs are compared between human and mouse.

To find the dysregulation associated with AD, we evaluated the co-expression changes in the brain of AD patients, which indicated the transcription regulation changes. The whole process is detailed in Figure 1. In the first step (a), four sets of independent human RNA-seq expression data were collected from the AMP-AD program compiling with the data access control policy. The selected samples from different projects were processed and combined together to define the expression profiles of both AD and control subjects [see step (b) and Figure S1]. Based on the clinical annotation provided by the data suppliers, 1045 AD samples and 622 control samples were determined and selected [see step (c)]. As showed in Figure S2, these samples have good homogeneity in their transcriptomic expression profiles and there is no clear outliers or batch effects. The mouse expression data were also collected from the GEO database (http://www.ncbi.nlm.nih.gov/geo/), where 931 samples from 20 microarray experiments were selected and processed to construct the mouse brain expression profiles.

In the next step (d), the co-expression level, described by Spearman's correlations, of all gene pairs were calculated for AD and control subjects, respectively. To check the robustness of calculated correlations, we performed random sampling and shuffling to original expression data. We found that the calculated values were tolerant to the sample selection and were less likely to result from random correlation (see Figure S3 and Methods). These results suggested the correlation value to be robust enough to study the co-expression changes. Applying the differential correlation analysis method introduced in Fukushima (2013), we found 87,539 out of 163 million gene pairs to have significant co-expression changes in the AD patients at a cutoff of adjusted *p* < 0.01 (see Table S1). Among them, there were 9,168 genes with at least one DCE partner (see Table S2). Considering the nature of DCE analysis and the strict cutoff, all of the predicted gene pairs were co-expressed in either AD patients or normal people or both at a cutoff of *p* < 0.01 that calculated using transformed correlation values (see Methods and Table S1), which confirmed that all the dysregulated gene pairs were potentially associated with the transcription regulation or co-regulation in the AD or normal samples. Similarly, the DCE pairs were supposed to have altered regulation in the AD patients. Therefore, we could call them dysregulated genes.

We checked the co-expression status of the 87,539 dysregulated gene pairs. 22,150 gene pairs (25.3%) were co-expressed only in AD samples while 10,707 pairs (12.2%) were co-expressed only in normal samples at a cutoff of *p* < 0.01. Other gene pairs were co-expressed in both AD and normal samples. Among them, 31,685 pairs (36.2%) have increased co-expression correlation in AD while only 9,694 pairs (11.1%) have decreased values. There were also gene pairs with reversed co-expression trend. For example, 6,459 negatively correlated gene pairs (7.4%) in normal subjects become positively correlated in AD patients. Vice versa, 6,844 gene pairs (7.8%) have the opposite changes. By summarizing the overall changes, we observed more gene pairs to have increased co-expression correlation in AD (2.6 times that of the gene pairs with decreased co-expression correlation), which suggested a strengthened transcriptional regulation in the AD patients.

### 3.2. Dysregulated AD Genes

In published works, at least 777 expressed genes have been reported to be associated with AD. We found that 479 of them were predicted with at least one dysregulated partner (see Table S3)(*p* = 2.5*e*−9 by Fisher's exact test). Among them, the MAP1B gene was predicted with the maximum number of partners (365 genes), of which 32 genes were also AD associated genes. We found that the partners of MAP1B had diverse functions and many of them were associated with AD progression, such as intracellular signaling cascades (42 genes, *p* = 3.7*e*−4 and *FDR* = 0.2), regulation of apoptosis (18 genes, *p* = 3.4*e*−3 and *FDR* = 0.2), neuron and dendrite development (5 genes, *p* = 4.13*e*−3 and *FDR* = 0.3) and RNA metabolic process (19 genes, *p* = 4.71*e*−3 and *FDR* = 0.3). This result confirmed that MAP1B, as a microtubule associated protein, had diverse involvement in neuron related biological processes (Ishitani et al., 2009; Maurin et al., 2009; Moritz et al., 2009; Villarroel-Campos and Gonzalez-Billault, 2014) and took important roles in the AD progression (Ulloa et al., 1994). Another example was the TREM2 gene, which was reported to get involved in the neuroimmunology of AD (Bouchon et al., 2000). We observed 4 partners, including SLA (DCE at *p* = 3.7*e*−10 and *FDR* < 0.01), HCLS1 (*p* = 2.5*e*−10), C3AR1 (*p* = 8.7*e*−10) and FCER1G (*p* = 1.3*e*−9), all of which were associated with inflammation related functions. Among the well-studied AD drug targets (Silva et al., 2014), we found their dysregulation, such as the amyloid precursor protein gene (APP, 136 partners), glycogen synthase kinase 3 beta (GSK3B, 34 partners) and BACE1 (8 partners), suggesting their transcriptional involvement in the progression of AD.

In Table 1, we showed 68 dysregulated AD genes, which were selected based on the number of their partners. To understand their biological involvement, we studied the enrichment of AD related genes in their partners. We found the partners of 48 dysregulated AD genes enriched with AD genes at a cutoff of *p* < 0.05, indicating their close relationship with AD progression. We also checked the transcriptional association of 68 dysregulated AD genes. We found 56 genes to be differentially expressed (*p* < 0.05) in the AD patients and the significance for such enrichment was *p* = 1.1*e*−27 by Fisher's exact test. Similarly, we found that the partners of 53 dysregulated AD genes were enriched with the differentially expressed genes (DEGs), confirming the transcriptional involvement of the dysregulated AD genes. Another investigation was to the aging related genes based on the annotation in our previous work (Meng et al., 2016). We found 21 out of 68 dysregulated AD genes to be aging genes and the significance for such enrichment was *p* = 6.9*e*−5. The partner genes of 39 dysregulated AD genes were also enriched with the aging genes at a cutoff of *p* < 0.05, confirming the association of the aging process with the progression of AD.

**Table 1:** The dysregulated AD genes.

**Gene**	**partners**	**DEG**	**p_(*DEGs*)_**	**AD**	**p_(*AD*)_**	**Aging**	**p_(*aging*)_**
MAP1B	364	Yes	9.2e-77	Yes	3.3e-05	Yes	5e-3
PSAP	337	No	0.03	Yes	0.6	No	0.01
PGAM1	300	Yes	1.4e-24	Yes	0.04	No	2e-3
ARID1B	275	No	4.8e-4	Yes	0.36	No	6.5e-11
SLC1A2	271	No	1.9e-40	Yes	6.1e-3	No	0.18
TGFBR2	243	Yes	1.1e-72	Yes	6.4e-06	No	1.9e-14
SREK1IP1	209	No	0.55	Yes	0.05	No	0.02
SYNJ1	200	Yes	6.3e-13	Yes	0.15	No	0.41
DOCK3	195	Yes	4.1e-11	Yes	0.01	No	0.22
STAT3	182	Yes	3.41e-49	Yes	1e-3	No	1.9e-11
SUCLA2	164	Yes	8.4e-13	Yes	0.49	Yes	ee-3
SERPINI1	153	Yes	5.9e-32	Yes	0.01	Yes	6.3e-4
AMPH	141	No	8.7e-22	Yes	0.4	No	0.19
APP	136	Yes	1.2e-11	Yes	0.23	No	0.38
CLU	130	Yes	0.24	Yes	0.66	No	0.02
LRP1	126	Yes	2.9e-16	Yes	0.09	No	2.9e-05
GABRG2	126	No	6.9e-11	Yes	0.63	No	0.18
VAT1L	121	No	7.9e-13	Yes	0.34	No	0.02
PHF1	119	No	0.4	Yes	0.59	No	2e-2
TAGLN3	113	Yes	6.2e-32	Yes	2.4e-07	No	5e-06
FRMD6	109	No	1.5e-21	Yes	0.04	No	3.4e-4
PDSS1	106	No	0.68	Yes	0.67	Yes	2.9e-4
PPIA	106	Yes	4.2e-45	Yes	0.01	Yes	7.34e-09
INPP5D	96	Yes	1.6e-27	Yes	2.3e-3	No	3.6e-06
PRKCE	94	Yes	4.3e-4	Yes	0.05	Yes	0.35
APLP2	94	No	7.4e-07	Yes	0.05	No	0.16
YWHAB	94	Yes	1.4e-17	Yes	0.34	Yes	3.6e-3
GABRG3	87	Yes	4.3e-11	Yes	0.96	No	0.74
PPARA	83	Yes	5.8e-31	Yes	2.9e-3	No	9.2e-05
RAN	83	Yes	0.03	Yes	0.36	Yes	9.4e-3
VDAC1	83	Yes	3.9e-10	Yes	0.28	Yes	0.02
AFF1	82	Yes	2.7e-24	Yes	0.13	No	5.9e-06
PFDN2	81	Yes	4.5e-16	Yes	0.13	Yes	1.5e-09
EBP	80	No	3.6e-23	Yes	6.7e-3	No	6.5e-08
LEMD2	78	No	0.01	Yes	0.82	No	4.7e-3
PRKACB	76	No	1.6e-3	Yes	0.53	Yes	0.02
EID1	70	Yes	0.33	Yes	0.21	No	1.3e-3
GSTO1	66	Yes	3.5e-26	Yes	0.01	Yes	1.1e-08
CNTNAP2	66	Yes	1.5e-08	Yes	0.16	No	0.55
GABRA3	65	Yes	6.8e-08	Yes	0.59	No	0.32
ARHGAP20	64	Yes	1.3e-3	Yes	0.21	Yes	0.15
PAK1	62	Yes	4.2e-09	Yes	0.61	No	8.6e-3
THOP1	62	No	0.34	Yes	0.03	Yes	0.36
AKT1S1	61	Yes	0.35	Yes	0.75	No	0.19
PRNP	60	Yes	4.1e-05	Yes	0.25	No	0.02
RTN3	60	Yes	7.3e-14	Yes	0.14	Yes	4.3e-3
STX2	60	No	0.94	Yes	0.73	No	0.22
GAS7	59	Yes	0.01	Yes	1	No	0.02
CCDC134	58	No	0.91	Yes	0.71	No	0.7
GRM8	58	Yes	4.2e-12	Yes	0.34	No	0.06
GAPDH	55	No	0.04	Yes	0.28	No	0.06
CHL1	53	Yes	4.1e-4	Yes	0.43	Yes	0.05
CHRM4	52	No	1.5e-09	Yes	0.56	No	0.07
GABRB2	50	No	5.3e-09	Yes	0.06	No	0.47
NEDD4	50	Yes	0.43	Yes	1	No	0.07
VSNL1	48	Yes	5.8e-05	Yes	0.48	No	0.12
PSENEN	47	No	3.9e-05	Yes	0.01	No	3.0e-3
BECN1	46	Yes	0.97	Yes	0.47	Yes	0.28
CHMP5	45	Yes	0.19	Yes	0.48	Yes	5.9e-4
UBQLN1	44	No	0.91	Yes	0.57	Yes	0.01
HECW1	44	No	0.04	Yes	0.21	No	0.94
CSMD1	44	Yes	3.2e-09	Yes	0.8	No	0.15
CHRM2	44	Yes	1.4e-3	Yes	0.48	No	0.08
UCHL1	43	Yes	2.8e-11	Yes	9.7e-3	No	0.01
PHYHD1	43	Yes	2.9e-3	Yes	0.85	Yes	0.07
SCN1A	41	No	0.11	Yes	0.8	Yes	0.37
APBA1	41	No	0.06	Yes	0.25	No	0.12
UQCR10	41	Yes	1.02e-05	Yes	0.03	No	7.9e-3

**Figure 2:**
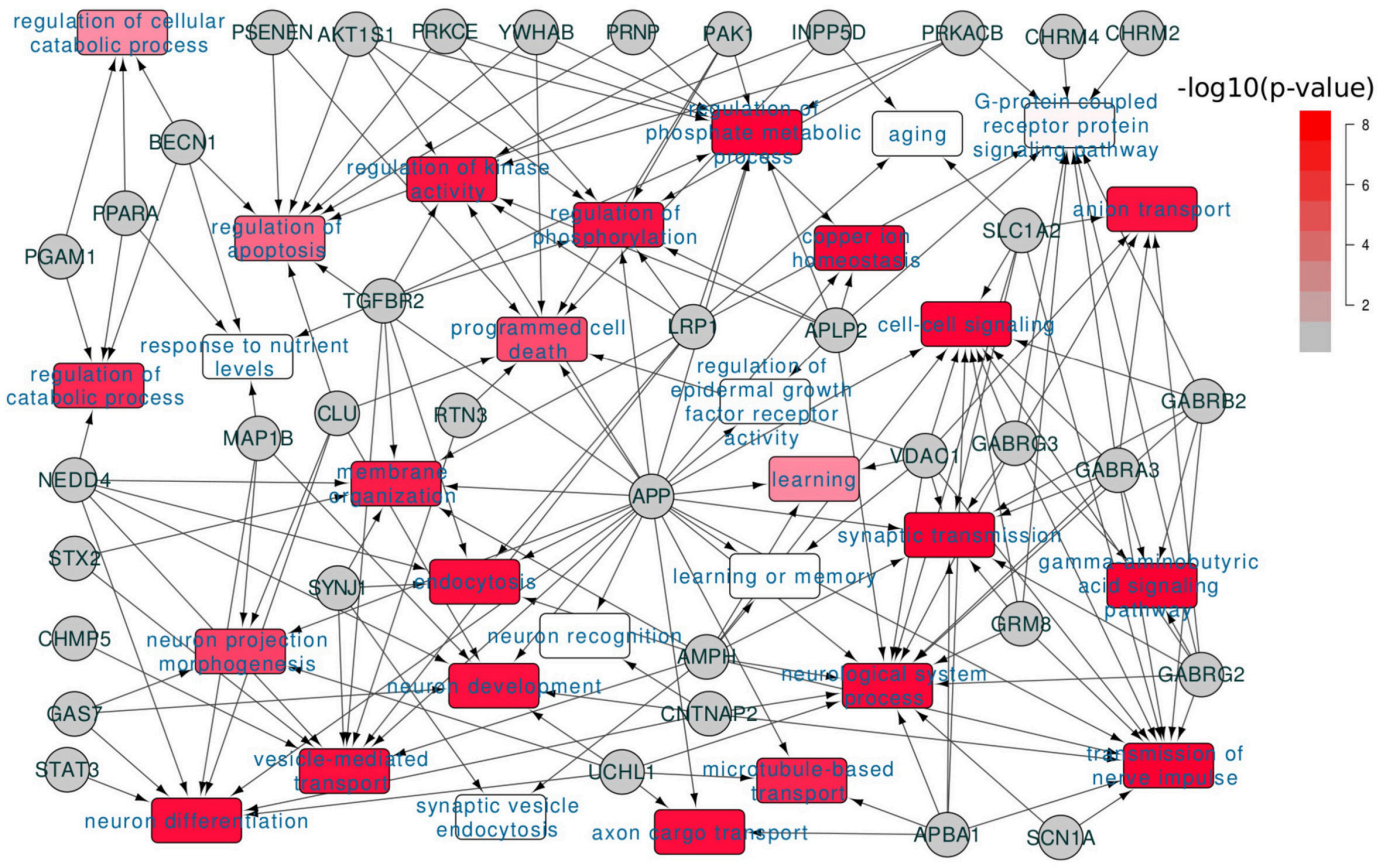
Functional involvement of dysregulated AD genes. 68 dysregulated AD genes were annotated for their functional involvement based on the Gene Ontology annotation; the colors of GO terms indicated the enrichment significance.

We also studied the functional involvement of the dysregulated AD genes. In Figure 2, we showed the functional annotation for the 68 dysregulated AD genes based on Gene Ontology annotation. Consistent with the selecting criteria, we found that these genes were associated with many AD related functions. Among them, “transmission of nerve impulse” was predicted to be the most enriched term (12 genes, *p* = 2.3*e*−7 and *FDR* < 0.05). In the published works, synaptic dysfunction had been widely reported for its association with AD (Selkoe, 2002; Pozueta et al., 2013) and our analysis confirmed that it was one of the most affected processes. Other AD related terms include “neurological system process” (14 genes, *p* = 1.54*e*−3), “regulation of protein kinase activity” (7 genes, *p* = 3.5*e*−3).

In summary, we found a subset of the AD related genes that were transcriptionally dysregulated in AD; these genes were involved in the progression of AD by affecting the AD related pathways or biological processes, e.g., synaptic transmission.

### 3.3. A Core Network Involves the Dysregulation of AD

Following the widely accepted hypothesis for hub genes in a network, we assume that the genes with more dysregulated partners will take more essential roles in the etiology of AD (Zhang and Horvath, 2005). Another assumption is that the dysregulated genes and their partners will participate in the same pathways or biological processes. Therefore, we can infer the function of dysregulated genes by analysis of their partners (Meng and Vingron, 2014).

**Figure 3:**
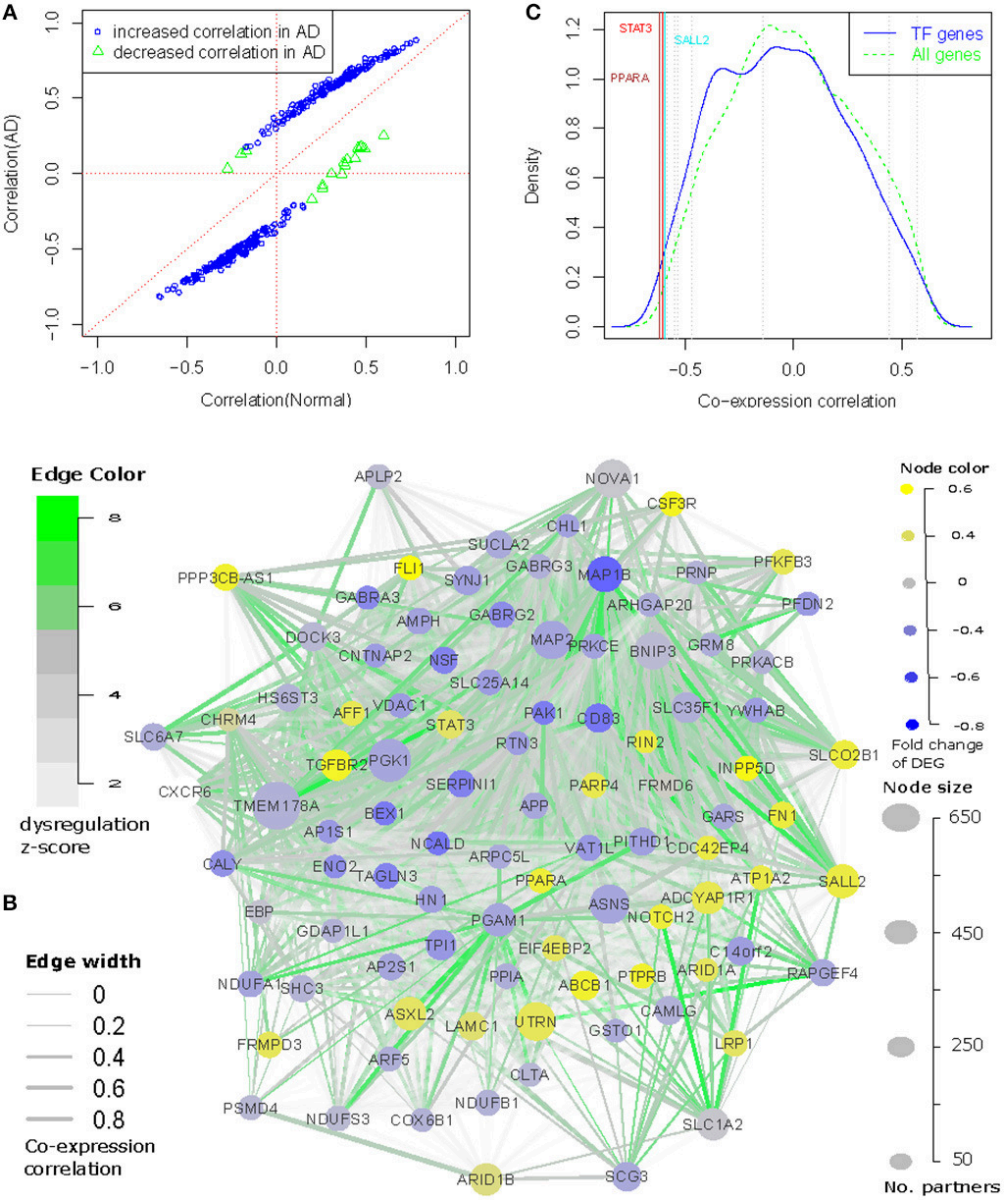
The core network involving the dysregulation of AD. We connected the dysregulated genes as a network and found that **(A)** Majority of the dysregulated genes have increased correlations (both direction) in the transmit from normal to AD; **(B)** 96 (out of 97) dysregulated genes can be interconnected as a regulatory network based on co-expression and differential co-expression information. **(C)** TMEM178A expression is negatively correlated with dysregulated transcription factors, among which PPARA is ranked as the most negatively correlated transcription factors.

Based on these assumptions, we defined the genes with both transcriptional dysregulation and involvement in the progression of AD by the following criteria: (1) with more than 50 partners; and (2) to be differentially expressed in AD (*p* < 0.01, see Table S4), which ensures the selected genes to be transcriptionally associated with AD, and their partners to be enriched with AD genes; or (3) reported as AD associated genes and their partners enriched with the differential expressed genes at a cutoff of *p* < 0.01. In this way, 97 dysregulated genes were selected (see Figures 1C,D and Table S2). Among them, 64 genes were differentially expressed in AD and 39 genes were reported as AD associated genes. These genes were dysregulated in 14,322 gene pairs with 3681 partners. Of the dysregulated genes, TMEM178A was the most dysregulated gene with 669 partners. We checked the co-expression status between dysregulated genes and their partners and found 85.9% of the gene pairs to have increased co-expression correlation, which was far more than the observed percentage (66.2%) with non-filtered dysregulated gene pairs (Figure 3A). In AD patients, we also observed 3461 gene pairs co-expressed only in AD, which was 3.6 times of the normal-specific co-expressed pairs (*p* = 4.6*e*−67 by Fisher's exact test), suggesting a strengthened transcriptional regulations in AD patients.

Even though the dysregulated genes were not selected for any co-expression correlation among each other, we observed the 97 dysregulated genes displaying stronger co-expression correlation values than randomly selected genes. Of 4,656 gene pair combinations, 95.7% of them were co-expressed at a cutoff of *p* < 0.01 and 86.9% have a correlation *r*>0.3. The median correlation value was 0.556 (*p* = 7.7*e*−86 by correlation test). We also checked their co-expression changes and found 929 out of 4,656 gene pairs to be differentially co-expressed in the transition from normal to AD.

Using the co-expression and differential co-expression information, we could connect the dysregulated genes as a network (see Figure 3B). In this network, 96 out of 97 nodes had at least one connected co-expression partner at *r* > 0.3 and 90 nodes can also have at least one dysregulation partner at a cutoff of adjusted *p* < 0.01. Combining the co-expression and differential co-expression information, 96 out of 97 dysregulated genes were connected with other dysregulated genes. The island of this network was ARF5, which was differentially expressed in AD but not reported with any association with AD. Therefore, we filtered it in Figure 3B. We further checked the direction of edges and found 870 out of 929 dysregulated edges to have increased co-expression correlation in AD while 176 of these regulations were AD-specific. In Figure 3B, we also showed the differential expression information of 96 nodes and found 26 up-regulated and 37 down-regulated genes in AD. The transcriptional regulations were observed between up- and down-regulated genes, including 42.7% of co-expressed edges to have negative correlation values.

In the network, we observed 10 transcription factors (TFs): FLI1, NOTCH2, SALL2, STAT3, PPARA, BEX1, ARID1A, ARID1B, AFF1, and PRNP. We checked if these 10 TFs regulated the expression of other dysregulated genes. Due to limitation for experimental validation, e.g., human brain sample collection and manipulation, we evaluated their regulatory roles by comparing their expression profiles with the dysregulated genes. Compared with 2405 annotated TF genes in the Gene Ontology, we observed that these TF genes were always ranked as the most co-expressed TF genes. In Figure 3C, we showed the example for TMEM178A gene. We found that 9 TFs had strong co-expression correlations, especially for PPARA and STAT3, which were co-expressed with TMEM178A at *r* = −0.62 and *r* = −0.603, ranked as the 10th and 16th of the most negatively co-expressed TF genes. Similar results were observed with other dysregulated genes (see Figure S4). Another investigation was to predict the gene-specific regulators using the method introduced in Meng and Vingron (2014). In this step, we predicted the enriched regulators for each of 97 dysregulated genes using AD and normal expression data as the input expression matrix. In the Jaspar database, only STAT3 had clear TF binding profiles and therefore, we could only predict the transcriptional regulation for STAT3. We found that STAT3 was ranked as the 16th of the most important regulators for 96 dysregulated genes in 474 annotated TFs. In normal and AD samples, STAT3 was predicted to regulate 25 and 31 dysregulated genes, respectively. Overall, these results suggest that the dysregulated TFs may be involved in transcriptional regulation or dysregulation in AD.

### 3.4. Aging, Synaptic Transmission and Metabolism Are Dysregulated

Applying a strategy of guilt by association, we extended the functional study of 97 dysregulated genes to the subnetworks comprising of themselves and their partners. In this step, we used each of 97 dysregulated genes as hub and exacted the partner genes to construct the dysregulation subnetwork, which could be treated as the co-regulated unit for further studies. Investigation of these subnetworks suggested them to have overall consistent co-expression changes. Taking the TMEM178A subnetwork as an example, we found 665 out of 669 nodes to have increased co-expression correlations with the hub gene, including 164 gained co-expression in AD. Similar results were observed with many other dysregulated genes (see Table S2).

**Figure 4:**
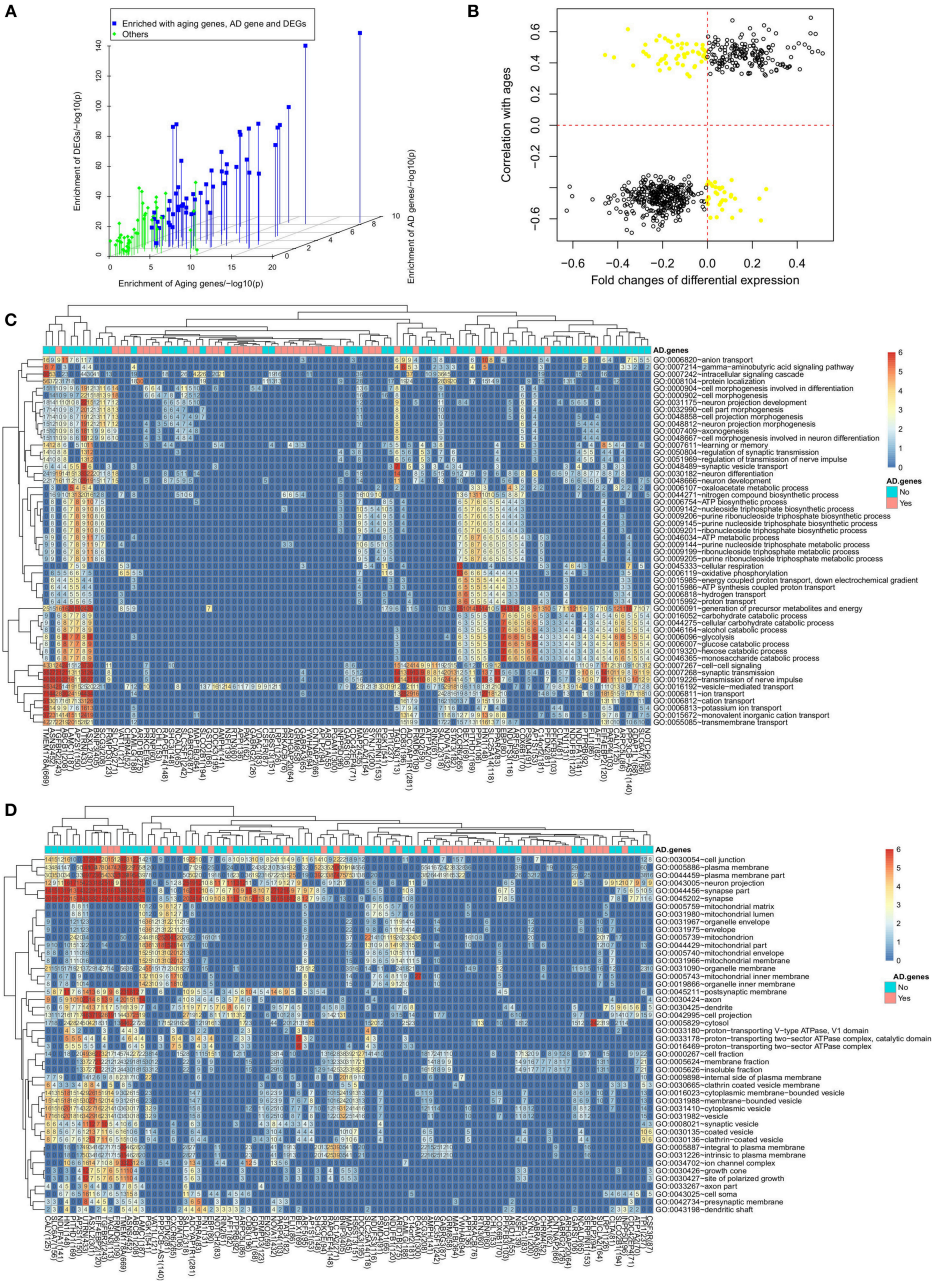
Aging, synaptic transmission and metabolism are dysregulated. **(A)** The enrichment of aging genes, AD differentially expressed genes and AD related genes in 97 subnetworks; **(B)** the dysregulated genes usually have the same expression change direction in the aging process and the AD progression; **(B)** functional enrichment analysis to dysregulated genes for **(C)** biological process and **(D)** cellular component.

We investigated the association of subnetworks with AD progression. The first attempt was to check the enrichment of DEGs in AD. We found all the subnetworks (97/97) to be enriched with DEGs at a cutoff of *p* < 0.01 (see Figure 4A). Taking the TMEM178A subnetwork as an example, 413 out of 669 partner genes was differentially expressed in AD and the significance for this enrichment was *p* = 6.5*e*−127 by Fisher's exact test. By checking the other genes, we found 84 subnetworks enriched with DEGs with a good statistical significance of less than *p* = 1*e*−10 by Fisher's exact test. Such significant enrichment suggested that all the subnetworks were associated with the progression of AD at the transcriptional regulation level.

Considering the fact that age is the biggest risk factor for AD, we checked the enrichment of aging related genes in each subnetwork which were identified by finding 2,862 genes with aging associated expression or DNA methylation in our previous work (Meng et al., 2016). For the subnetworks with differentially expressed genes as hubs, we found 53 out of 64 subnetworks significantly enriched with aging related genes (see Figure 4A). Taking the TMEM178A subnetwork as an example, we found 165 out of 669 genes to be aging related genes (*p* = 3.2*e*−20 by Fisher's exact test). Out of 2,862 aging genes, 624 genes were also dysregulated in AD. We further evaluated the involvement of dysregulated aging genes in the progression of AD by checking their differential expression direction in AD and their expression trend in the aging process. As showed in Figure 4B 541 dysregulated aging genes had the same expression direction, e.g., the aging related genes with increased expression levels in the aging process would be up-regulated in the AD patients and vice versa. Overall, the analysis results suggested that the aging genes were associated with the transcriptional dysregulation of the AD progression.

We performed functional annotation using David (Sherman et al., 2007) for each subnetwork. Figure 4C showed the enriched biological processes (BPs). The most enriched category was the synaptic function related terms. One example was the “transmission of nerve impulse” term, which was enriched in 37 subnetworks and that was also the most enriched term. The other related terms include “synaptic transmission” and “cell-cell signaling,” which had been reported for their involvement in the AD progression (Selkoe, 2002; Pozueta et al., 2013). Another category was the metabolism related terms. Among them, “generation of precursor metabolites and energy” was significantly enriched in 39 subnetworks and “glycolysis” was significantly enriched in 29 subnetworks. The links between metabolism and AD were also supported by the published works (Vanhanen et al., 2006; Suzanne and Tong, 2014). Even as different functional categories, synaptic function and metabolism related terms were usually enriched by the same subnetworks. We also observed other enriched terms and most of them had been reported for association with AD, such as “cation transport” (Berridge, 2014), “ATP metabolic process” (Liu et al., 2013), “learning or memory” (Liu et al., 2013) and “neuron differentiation.” Using David, we also checked the cellular component (CC) enrichment (see Figure 4D). We found neuron, especially synapse related CCs to be the most enriched cellular location. Consistent with BP analysis results, the “synapse part” term was enriched in 46 subnetworks, especially the subnetwork with dysregulated DEGs as hubs confirming the analysis results with BP terms.

As described above, the dysregulated genes were either DEGs or literature reported AD genes. We found that the functional involvement for the two groups of subnetworks was different. The 39 subnetworks with hubs of AD genes were less associated with any enriched biological process. For example, the term “transmission of nerve impulse” was enriched in only 8 out of 39 subnetworks while there were 29 out of 64 DEG-hub subnetworks enriched with this term. Similarly, the other AD associated terms were also less likely enriched with these 39 subnetworks. However, considering the fact that the dysregulated AD genes themself were associated with the AD related terms (as discussed in above section), including synaptic function and metabolism related biological processes, we could assume that these 39 subnetworks with the hubs of dysregulated AD genes were also associated with the enriched terms.

### 3.5. Association With Clinical Outcomes

In the ROSMAP project of AMP-AD program, most samples have been annotated with clinical information. We studied the association of gene expression with three disease related clinical traits, e.g., cognitive test scores (cts), braak stage (braaksc) and assessment of neuritic plaques (ceradsc). We did not find any gene to have strong expression correlations (e.g., *r* > 0.6) with studied traits. The maximum correlation was observed with SLC6A9 at *r* = −0.388. For all the genes, only 12.3% of them have a clinical association at |*r*| > 0.15. This result suggests that the clinical outcomes of AD may be affected by combined effects and even beyond the effects of gene expression.

**Figure 5:**
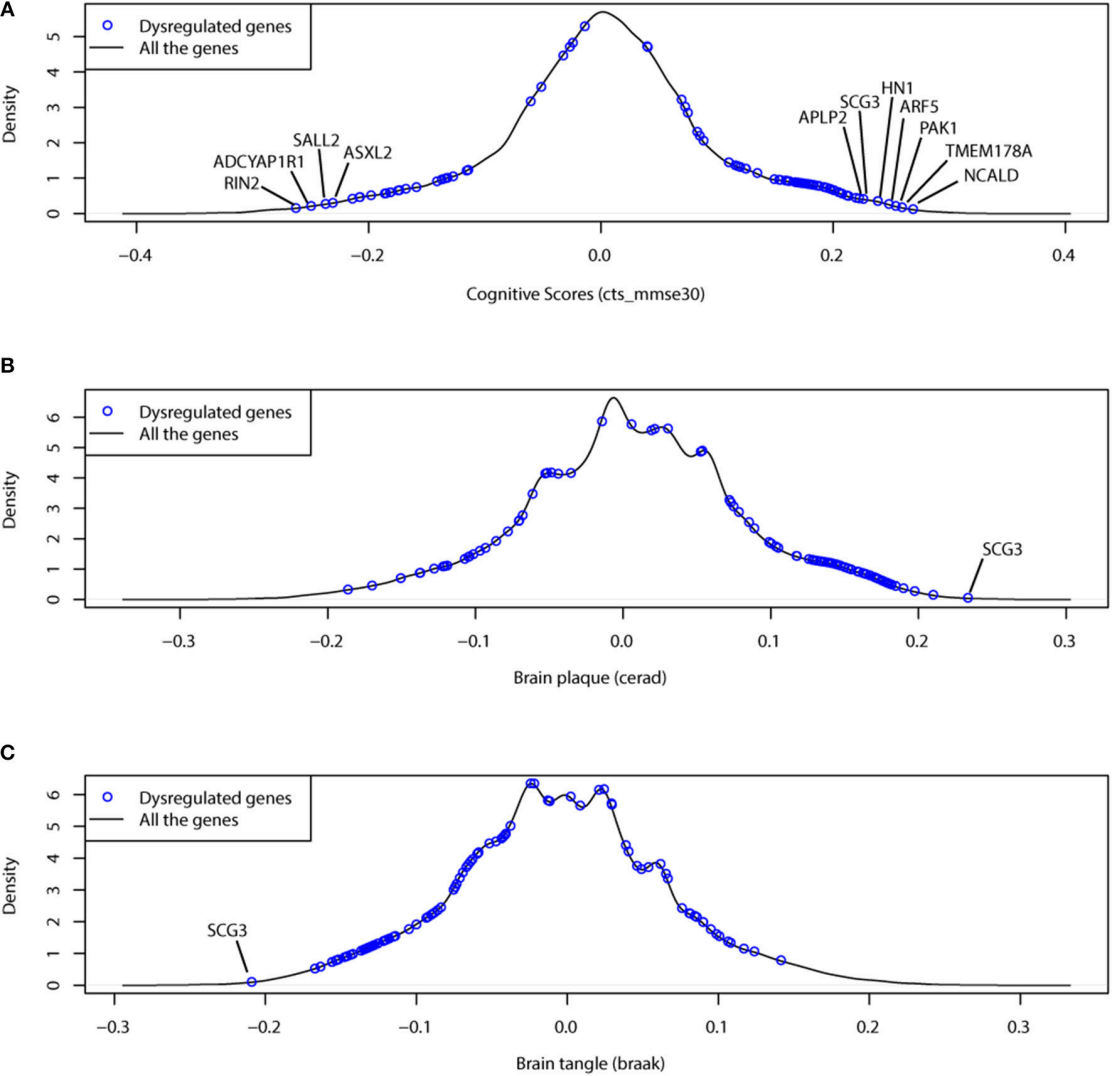
The dysregulated genes prefer to have more association with clinical traits, including **(A)** cognitive test score (cts), **(B)** braak stage (braaksc) and **(C)** assessment of neuritic plaques (ceradsc). The black lines show the density plots of clinical association (correlation) for all protein coding genes; the blue points indicate the 97 dysregulated genes.

In Figure 5, we showed the clinical association results for 97 dysregulated genes. We found that the dysregulated genes were always ranked as the most clinical associated genes. Of 97 dysregulated genes, 66 genes had a clinical association of |*r*| > 0.15 (*p* = 4.49*e*−37 by Fisher's exact test). Among them, NCALD, a neuronal calcium binding protein, was associated with the cognition score at *r* = 0.268.

Another investigation was to the combined effects of dysregulated gene pairs. We studied the partial correlations between the dysregulated genes and the clinical traits by controlling the effects of their dysregulated partners. We found that many dysregulated gene pairs had improved clinical association (see Table 2 and Table S5). In these dysregulated gene pairs, the clinical association of one gene was improved by considering the gene expression of its partners, which indicates the importance of dysregulation relationship.

**Table 2:** The association of dysregulated genes with the cognitive test scores.

		**coexpression cor**.		**cor. with cts^a^**		**partial cor.^b^**	
**Gene1**	**Gene2**	**Ad**	**Normal**	**Gene1**	**Gene2**	**Gene1**	**Gene2**
RPS19	APLP2	–0.446	–0.696	–0.036	0.226	0.205	0.299
SLC35F1	PPP2CA	0.718	0.528	0.039	0.208	–0.183	0.271
SLC35F1	RTN3	0.711	0.517	0.039	0.217	–0.180	0.273
RALGPS1	APLP2	–0.039	0.265	0.030	0.226	–0.188	0.289
SLC1A2	FAT1	0.233	0.511	–0.052	–0.185	0.153	–0.232
SLC15A2	SLC1A2	0.442	0.693	–0.208	–0.053	–0.298	0.214
SDC2	SLC1A2	0.359	0.611	–0.199	–0.053	–0.268	0.180
NOTCH2	SLC1A2	0.172	0.533	–0.210	–0.053	–0.267	0.177
SYNJ1	PRUNE2	0.333	0.581	0.160	0.062	0.218	–0.163
GPRC5B	SLC1A2	–0.055	0.27	–0.276	–0.053	–0.316	0.169
SUCLA2	PRUNE2	0.311	0.557	0.153	0.062	0.210	–0.158
SUCLA2	ARFGEF2	0.746	0.861	0.153	0.067	0.201	–0.148
PRKACB	CDS2	0.641	0.794	0.187	0.070	0.225	–0.145
PRKCE	KCNA1	0.62	0.397	0.196	0.062	0.237	–0.148
APP	RBMX2	–0.037	–0.353	0.169	–0.064	0.195	0.118

a *The correlation between gene expression and cognitive test score*.

b *The partial correlation with cognitive test score*.

### 3.6. Dysregulation Divergence in Mouse

**Figure 6:**
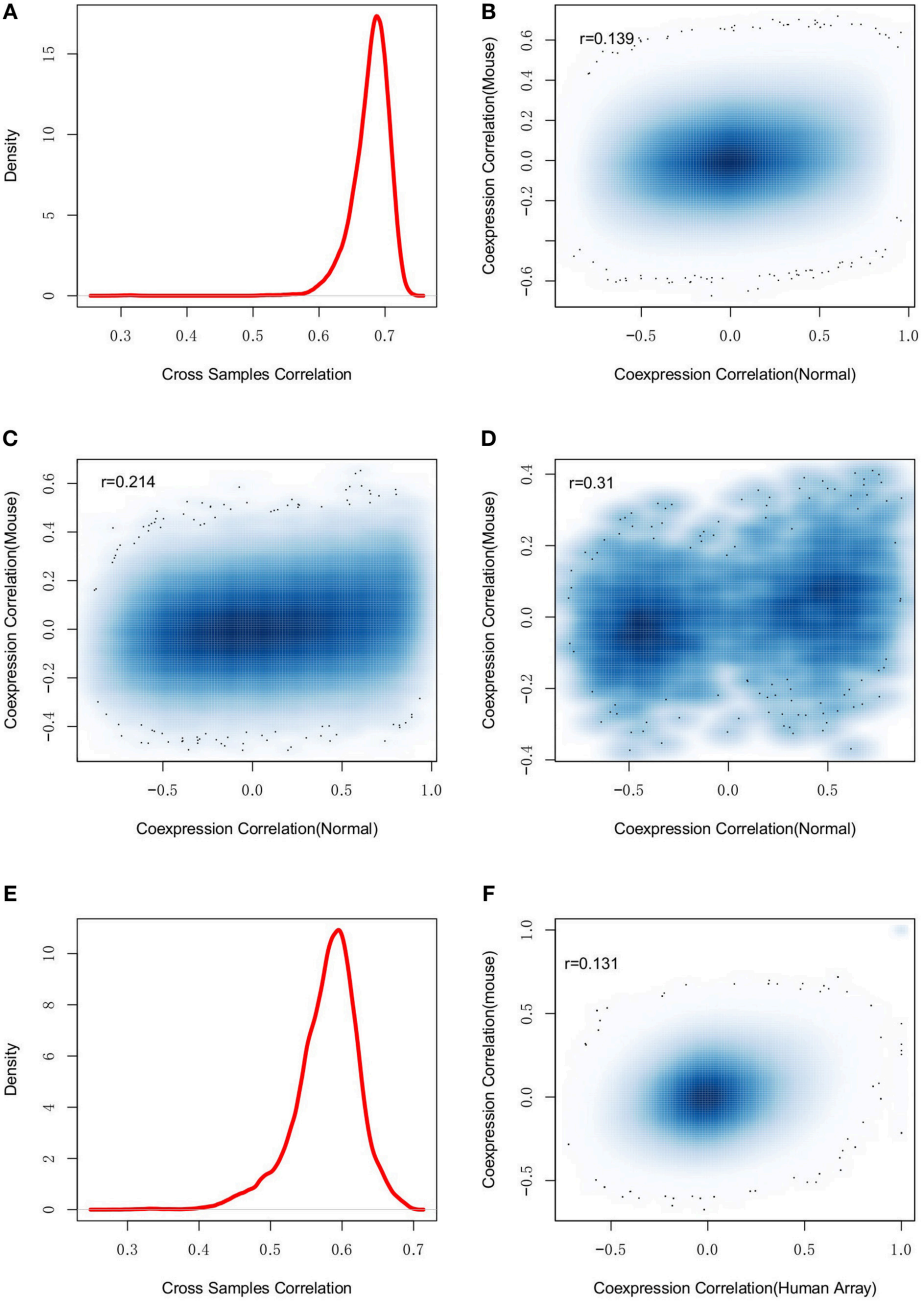
The dysregulation divergence between human and mouse. **(A)** shows the density plot of expression profile similarity (correlation) of human and mouse homologous genes, which suggests the conserved gene expression patterns between human and mouse. However, **(B)** the regulations among all the genomic genes are not conserved between human and mouse (*r* = 0.139). By restricting the studied regulations to the dysregulated pairs **(C)** and the edges in Figure 3 **(D)**, the conservation between human and mouse regulations was weakly improved. The observation was further investigated with human microarray data and found good consistent with human RNA-seq **(E)** but still failed to suggest any gene-gene regulation conservation between human and mouse **(F)**.

Mice are widely used as a model animal in wet-lab studies for AD. Investigation of the evolutionary conservation of gene regulation can help with the evaluation of experimental studies performed in mice. Therefore, we collected mouse brain microarray expression data from GEO database. One thousand five and hundred eighty three samples from 24 experiments were identified (see Table S6). We removed arrays with poor quality or inconsistent expression profiles and finally, 931 samples from 20 experiments were used. The selected samples were combined together to describe the mouse brain expression profiles. Using gene homologous annotation from Mouse Genome Informatics (MGI) (www.informatics.jax.org), 14,186 expressed genes were used for co-expression evaluation with the human normal control samples. We first studied the overall gene expression similarity between mouse and human. As showed in Figure 6A, we found human and mouse to have consistent expression profiles with the median Spearman's correlation among samples at *r* = 0.69, indicating the conservation of gene expression profiles between human and mouse.

Another investigation was to compare co-expression profiles of gene pairs. The co-expression correlations of all gene pairs were calculated for mouse and human normal samples, respectively. Figure 6B showed the co-expression correlation values. Human and mouse had different co-expression profiles and the Spearman's correlation to co-expression correlations was only *r* = 0.139. Further, we performed differential co-expression analysis and found 25.0% of gene pairs to have differential co-expression at the cutoff of adjusted *p* < 0.01. We also checked the co-expression directions and found 34.7% of 10 million co-expressed gene pairs to have inconsistent correlation directions, indicating the divergence of co-expression patterns between human and mouse. Similar results were also observed with the human AD samples (see Figure S5). To understand the co-expression conservation of AD related genes, we restricted the same analysis to the 87,539 differential co-expressed gene pairs. As shown in Figure 6C, improved conservation was observed (*r* = 0.214, *p* = 6.46*e*−266). We further restricted the analysis to 97 dysregulated genes and found the co-expression conservation were weakly improved (*r* = 0.31, *p* = 8.08*e*−6) (see Figure 6D).

Different platforms, e.g., RNA-seq and microarray, may lead to different expression measurements (Giorgi et al., 2013; Zhao et al., 2014). Therefore, we constructed the human brain expression profiles by collecting the microarray data from GEO database (see Table S7). To minimize the influences of different platforms, we only collected data from AffyMetrix's platform when the mouse expression data were also generated using AffyMatrix's technology. Finally, 452 samples were selected to describe the human brain expression profiles. As showed in Figure 6E, we found that the expression profiles measured by microarray and RNA-seq were not completely consistent (*r* = 0.54) (see Figure 6E). Next, we re-evaluated the co-expression conservation using the co-expression profiles calculated from human brain microarray data. The Spearman's correlation to the co-expression correlations was *r* = 0.152 (see Figure 6F), which still indicated strong dysregulation divergence between human and mouse.

In summary, human and mouse may have the divergent co-expression patterns in brain, even though they have the conserved gene expression profiles, which indicates the different transcriptional regulation.

### 3.7. Comparison With Established Works

**Figure 7:**
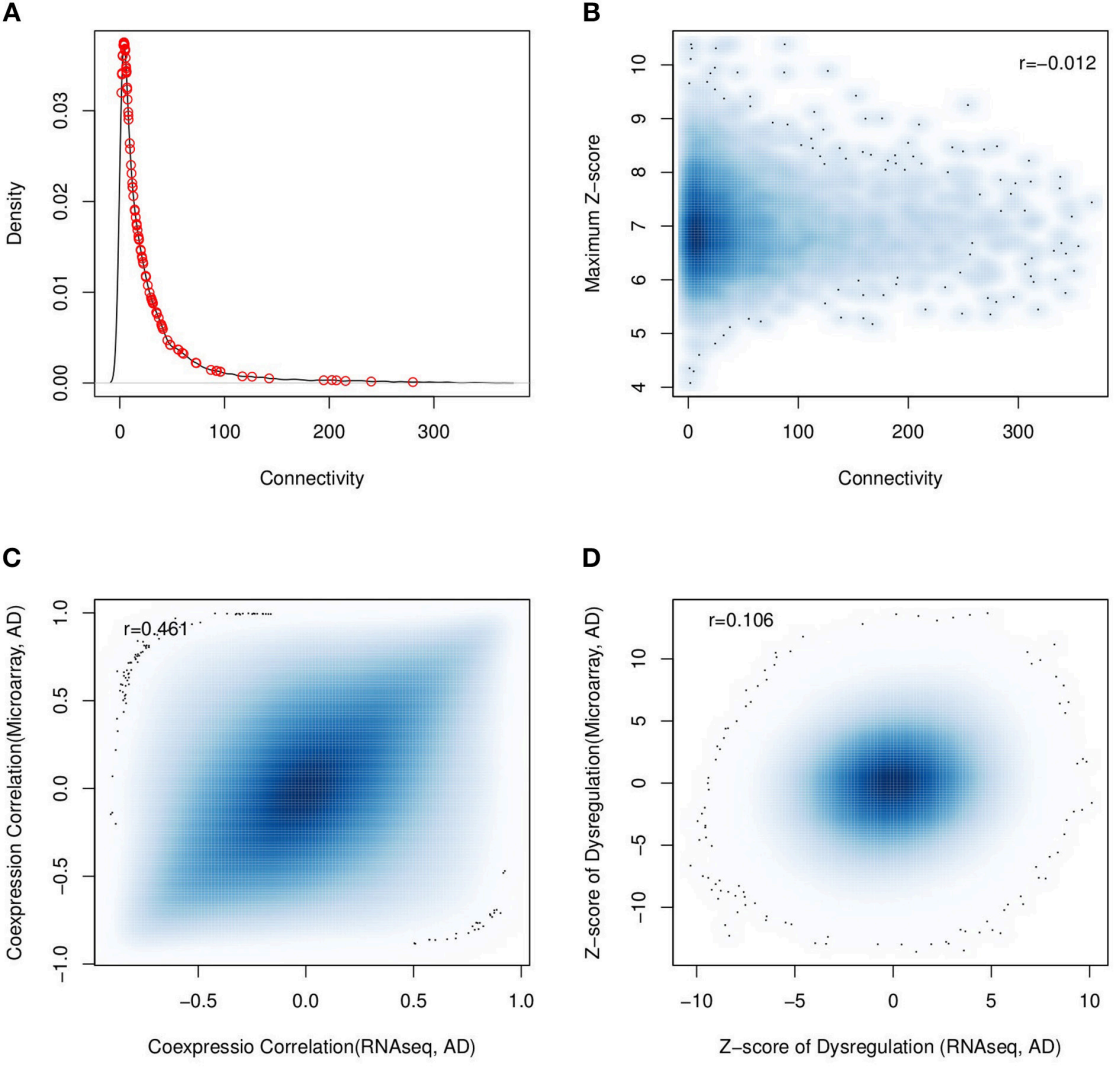
Comparison analysis with the results from established work. **(A)** The connectivity distribution of top 100 of the most dysregulated genes (red circle points) fails to indicate any association between connectivity and dysregulation; **(B)** the same result is observed with all the genomic genes (*r* = −0.012). **(C)** using human RNA-seq and micorarray data, the calculated gene co-expression correlations are consistent; but **(D)** the predicted dysregulation are not consistent.

Based on the assumption that the connectivity in the co-expression network indicates the gene importance, connectivity has widely been used to identify the essential components for a specific biological process. We investigated the association between dysregulation and the connectivity in the co-expression network. We firstly checked the connectivity preference of dysregulated genes by selecting the top 100 dysregulated genes discovered in the above analysis. As shown in Figure 7A), we failed to observe any connectivity preference when compared with genomic genes (*p* = 0.229). Another investigation was to check if the genes with stronger connectivity would be more differentially co-expressed. As shown in Figures 7B and Figure S6, the maximum and median differential correlation did not show strong association with the connectivity, including the most dysregulated ones.

Next, we investigated if the dysregulated genes could be identified by co-expression network analysis. We applied WGCNA (Langfelder and Horvath, 2008), a popular co-expression network analysis tool, to human brain RNA-seq expression data and 43 modules were predicted. The 97 dysregulated genes were mapped into 22 modules. We found that the number of dysregulated genes in a module was correlated with its module size (*r* = 0.76). For example, the BLUE module had the largest module size of 7,776 genes and it was also mapped with the maximum number of dysregulated genes (44 genes) (see Table S8). Further, we ranked the module members based on their connectivity in the modules. None of 97 dysregulated gene was ranked as the most connected hubs. All these results suggested that the dysregulated genes were relatively novel to hub genes in co-expression network analysis.

In independent work, the dysregulation of AD has been investigated using microarray data (Narayanan et al., 2014), where the dorsolateral prefrontal cortex region from 310 AD patients and 157 normal subjects was measured by microarray. Considering the fact that platforms may affect the analysis results (Giorgi et al., 2013), we compared their analysis results using 13,606 common expressed genes. The gene pairs from microarray and RNA-seq platforms had overall consistent co-expression profiles (*r* = 0.49, *p* = 1.2*e*−199) (see Figure 7C). Applying the differential correlation test, we found 18.5% of gene pair combinations to be differentially co-expressed, which was far more than the number of predicted dysregulations for AD progression: that was, 0.05% of all gene pairs for RNA-seq dataset and 0.6% for microarray datasets. This observation suggested that the dysregulations predicted using microarray and RNA-seq data were different. To illustrate it, we checked the consistency of predicted dysregulation by DCE analysis for both RNA-seq and microarray data. Figure 7D showed the dysregulation z-scores of all gene pairs, which described the significance of co-expression changes. We found that the overall dysregulation was weakly consistent between RNA-seq and microarray (Spearman's *r* = 0.106). At a cutoff of adjusted *p* < 0.01, 59,710, and 603,335 gene pairs were predicted to be dysregulated with RNA-seq and microarray data, respectively. Among them, only 1,469 gene pairs were shared by two datasets. We also checked the predicted partner number of dysregulated genes from RNA-seq and microarray data and found them to be weakly consistent (*r* = 0.218) (see Figure S7).

## 4. Discussions

Using the RNA-seq data, we studied the association between gene expression correlation and connectivity. We found that the genes with higher connectivity showed stronger co-expression correlations with other genes (see Figure S8A). Functional annotation to top 200 of the most connected genes suggested these genes more associated with house-keeping related roles, such as protein location and protein transport see Figure S8B. Considering the fact that AD is associated with loss of neuron related functions, we further checked the connectivity of 2,155 brain tissue-specific genes (Benita et al., 2010). Even though the brain-specific genes have stronger connectivity than the genomic genes (*p* = 7.53*e*−56), we found that only 14.8% of the brain-specific genes were ranked in the top 1,000 of the most connected genes. Similar results were observed with the AD related genes. All these observations suggested that the genes associated with AD progression were not necessary to have strong connectivity in co-expression analysis and this led us to extend the connectivity-based analysis to a less biased investigation.

We evaluated the co-expression differences between AD and normal samples for all the gene pair combinations without considering gene connectivity. The genes with differential co-expressed profiles were supposed to be dysregulated in the progression of AD. Using a similar assumption, the genes with more dysregulation partners were supposed to take more essential roles. Combining AD-related information, 97 dysregulated genes were identified and validated to take critical roles in the AD progression. Consistent with our hypothesis, the predicted genes failed to show strong connectivity in co-expression network analysis. This was further supported by mapping these genes into the WGCNA analyses results. These results suggested that this work revealed the etiology of AD in an independent and novel way.

We performed functional annotation on the 64 dysregulated DEGs and found them to take diverse functions. The limited gene number and the relative independence of dysregulated genes hindered the efficiency of functional annotation. We extended the functional studies of dysregulated genes to the investigation to their dysregulated partners. We observed 33 of them to be associated with synaptic transmission related functions while synaptic dysfunction was widely accepted as a key characteristic of AD progression (Pozueta et al., 2013). Evaluation using aging related genes indicated their wide and consistent involvement, which might explain the effects of age on AD progression. Overall, our analysis results suggested the predicted dsyregulation closely associated with the AD progression by affecting many well-reported mechanisms.

We defined the transcriptional “dysregulation” by selecting gene pairs with changed co-expression between disease and normal samples. The gene pairs could be down- or up-stream members in regulatory pathways, i.e., either A regulates B or B regulates A. They can also be co-regulated genes by common upstream pathways. In the context of co-expression or differential co-expression, it was not easy to elucidate the exact information of their relationship. However, by checking the functional annotation to the hub genes and their partners, we always found consistent functional annotation. For example, EIF4EBP2 was reported as a translation initiation repressor and got involved in synaptic plasticity, learning and memory formation (Bidinosti et al., 2010). Functional enrichment analysis to its partners identified the GO terms related to synaptic transmission, which confirmed the consistent functional involvement between hub genes and their partners.

This study is to find the correlation difference between AD and normal sample. Its confidence is highly dependent on the sample size (Fisher, 1915). Therefore, most of co-expression analysis studies recommend to include as many as possible samples and conditions (e.g., see Lee et al., 2004; Cahan et al., 2014; Gillis et al., 2015). This may lead to information loss, e.g., brain region-specific dysregulation. However, this also leads to identification of the consensus dysregulation that is more associated with AD progression. In differential co-expression analysis results are subtle and related to the batch or platform effects of the data from independent projects. We applied several steps to improve the confidence. The most critical one was to collect large-scale RNA-seq data from the AMP-AD program, which minimized the effects of different platforms and batches. Due to the large-scale RNA-seq data, we were able to perform deep evaluation of the quality of expression data by comparing the sample consistency.

Another novel finding is the divergent transcriptional regulations between human and mouse. As the widely used animal model, mouse is widely used in many neurological studies. Our finding puts a potential warning to some wetlab conclusions especially these from transcriptional related studies.

## Data Availability

The results published here are in part based on data obtained from the AMP-AD KnowledgePortal (doi: 10.7303/syn2580853) (see Doc S1 for a full acknowledgement to the individual data contributors).

## Author Contributions

GM conceived of the study, designed the study, carried out the analysis and drafted the manuscript. HM collected the data, conceived of the study and participated in its design and drafted the manuscript. All authors read and approved the final manuscript.

### Conflict of Interest Statement

GM and HM were employed by GSK R&D shanghai.

## Supplementary Material

Figure S1 The summarized sample information based on the annotation in AMP-AD projects, including **(a)** AD samples and **(b)** normal samples.

Figure S2 Genomic gene expression profile homogeneity evaluation for the samples from independent RNA-seq projects. The sample distribution in the principal component analysis (PCA) plot indicates the expression similarity of the selected samples for AD **(a)** and normal samples **(b)**, respectively.

Figure S3 Evaluation of the co-expression correlations by randomly sampling **(a)** and shuffling **(b)**. **(a)** Half of the sample are randomly selected to calculate the new correlations. **(b)** All the genes are shuffled with random samples annotation so that the gene pairs have the wrong sample mapping.

Figure S4 The co-expression between four TF genes and dysregulated genes. The solid lines indicate the co-expression correlation distribution between dysregulated gene and 2045 TF genes; the dashed line indicates the co-expression correlation between dysregulated genes and the genomic genes; the legend shows the TF genes with maximum co-expression correlation with dysregulated genes.

Figure S5 Mouse may have divergent co-expression profile with human AD samples.

Figure S6 The association between dysregulation and connectivity in the co-expression network. The y-axis shows the median z-score of the differential co-expression.

Figure S7 The partner number of dysregulated genes predicted using RNA-seq and microarray data.

Figure S8 The association between connectivity and co-expression correlation. **(a)** the genes with higher connectivity are always the genes with higher co-expression correlation. **(b)** Functional annotation to the top 200 genes with the highest connectivity.

Table S1 The 87,539 dysregulated gene pairs between AD and normal.

Table S2 All the dysregulated genes with at least one partner. Other annotation including differentially expressed in AD samples, aging related genes, AD genes, connectivity in co-expression network.

Table S3 The Alzheimer's disease related genes collected by text mining to the published works.

Table S4 The differentially expressed genes in AD patients.

Table S5 The association between dysregulated genes and clinical traits.

Table S6 The used microarray data for mouse brain.

Table S7 The used microarray data for human brain.

Table S8 The dysregulated genes and the WGCNA analysis results.

Doc S1 The full acknowledgement to the individual data contributors.
