# Supplementary figures and images for: Transcriptional Dysregulation Study Reveals a Core Network Involving the Progression of Alzheimer's Disease
Source: Front Aging Neurosci. 2019 May 7;11:101. doi: 10.3389/fnagi.2019.00101 (PMC6513962; doi:10.3389/fnagi.2019.00101)

Summarized clinical annotation of samples

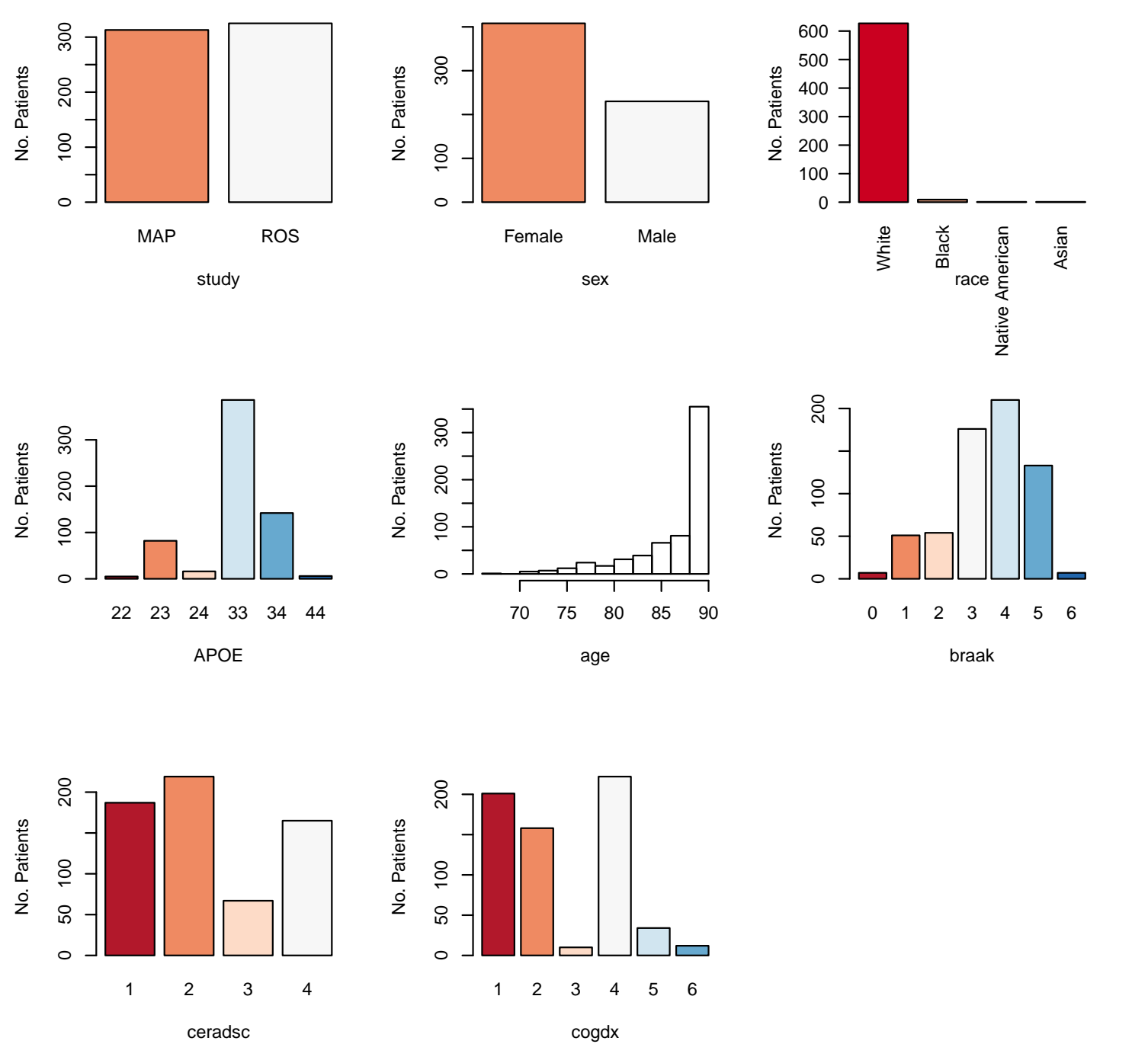

(a) RosMap

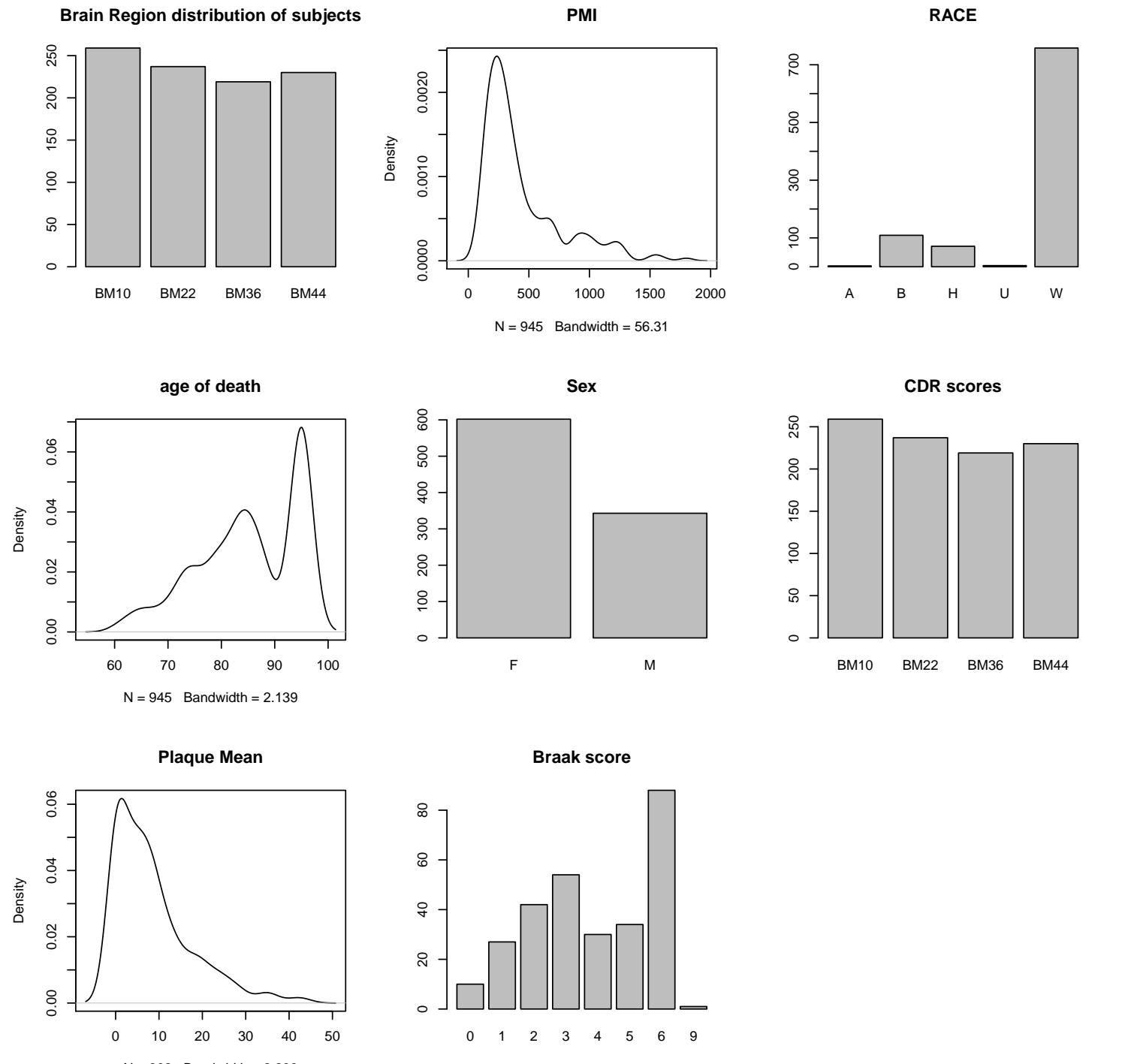

(b) MSBB

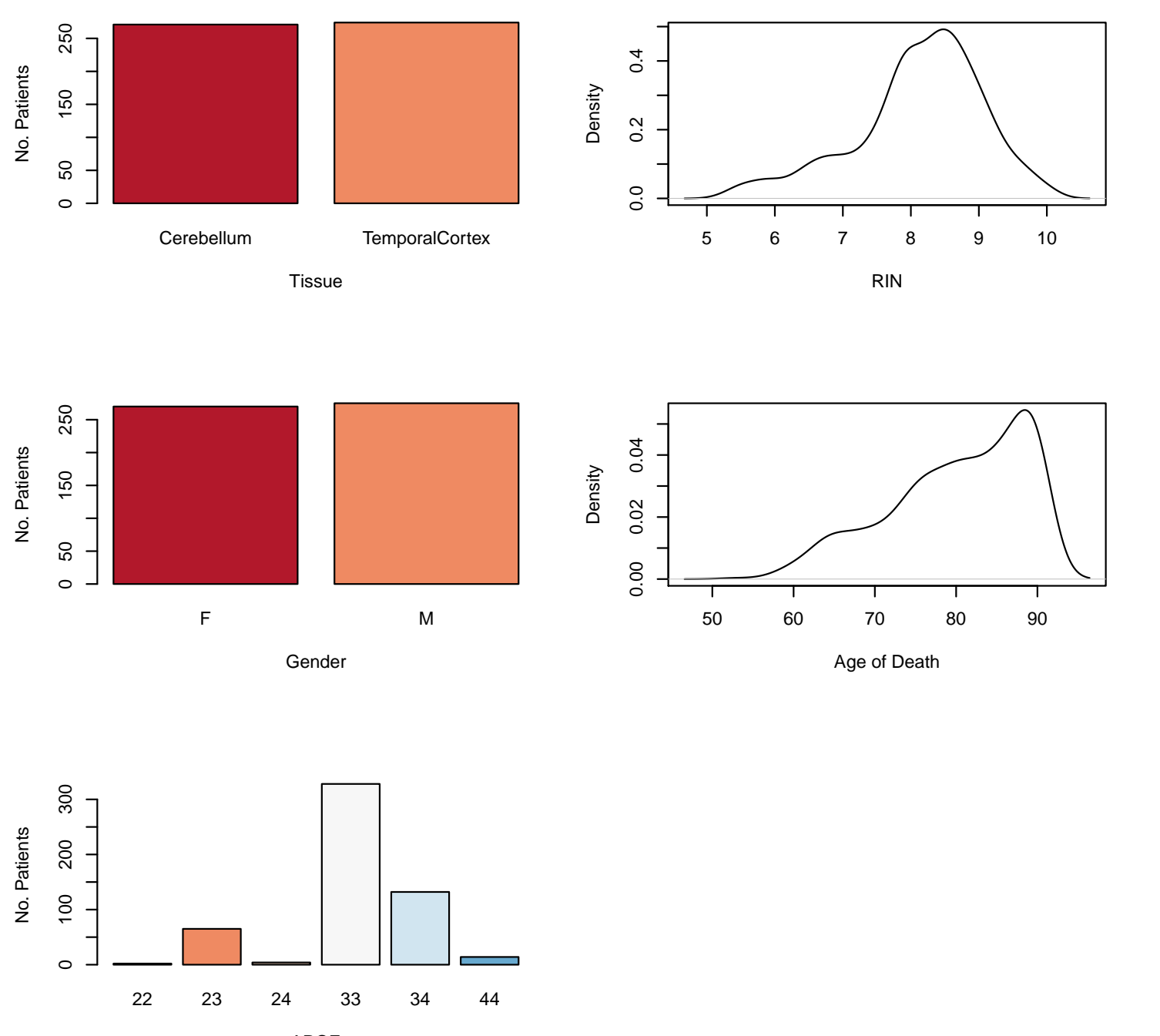

(c) Mayo

Supplement: Figure S1 — The summarized sample information based on the annotation in AMP-AD projects, including (a) AD samples and (b) normal samples. [file Data_Sheet_1.PDF]

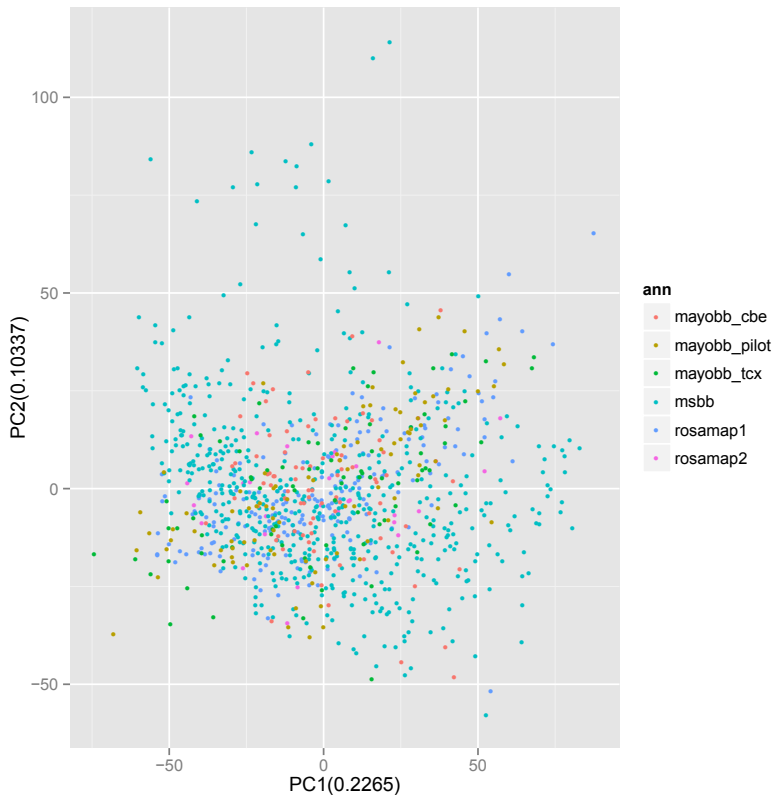

(a) AD samples

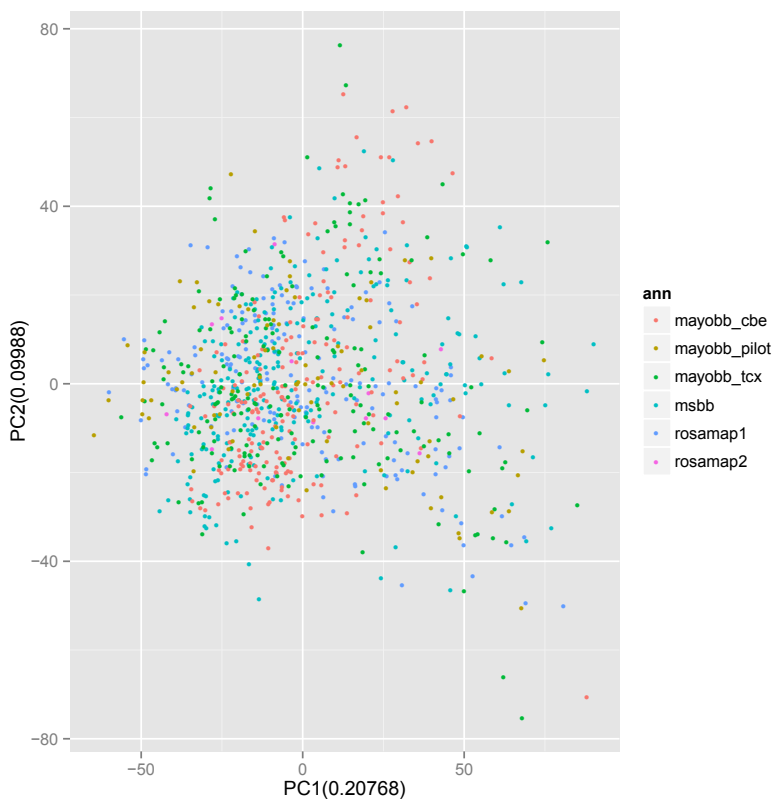

(b) Normal samples

Supplement: Figure S2 — Genomic gene expression profile homogeneity evaluation for the samples from independent RNA-seq projects. The sample distribution in the principal component analysis (PCA) plot indicates the expression similarity of the selected samples for AD (a) and normal samples (b), respectively. [file Data_Sheet_2.PDF]

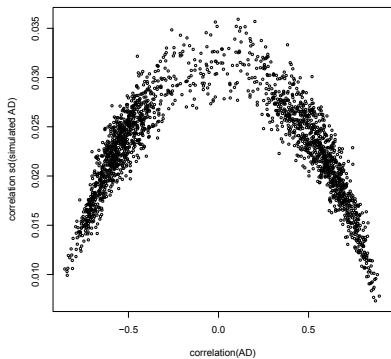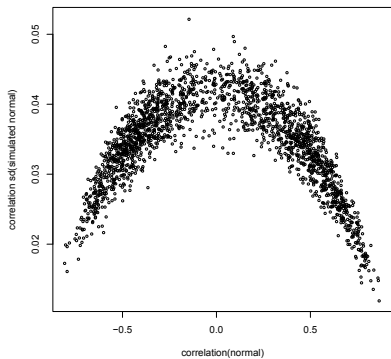

(a)

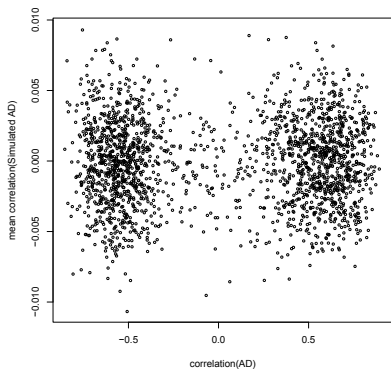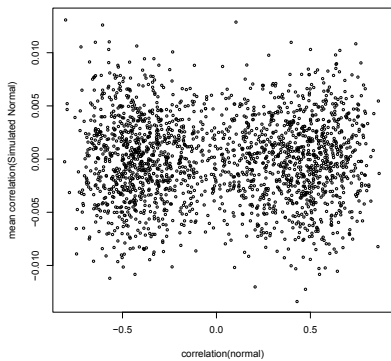

(b)

Supplement: Figure S3 — Evaluation of the co-expression correlations by randomly sampling (a) and shuffling (b). (a) Half of the sample are randomly selected to calculate the new correlations. (b) All the genes are shuffled with random samples annotation so that the gene pairs have the wrong sample mapping. [file Data_Sheet_3.PDF]

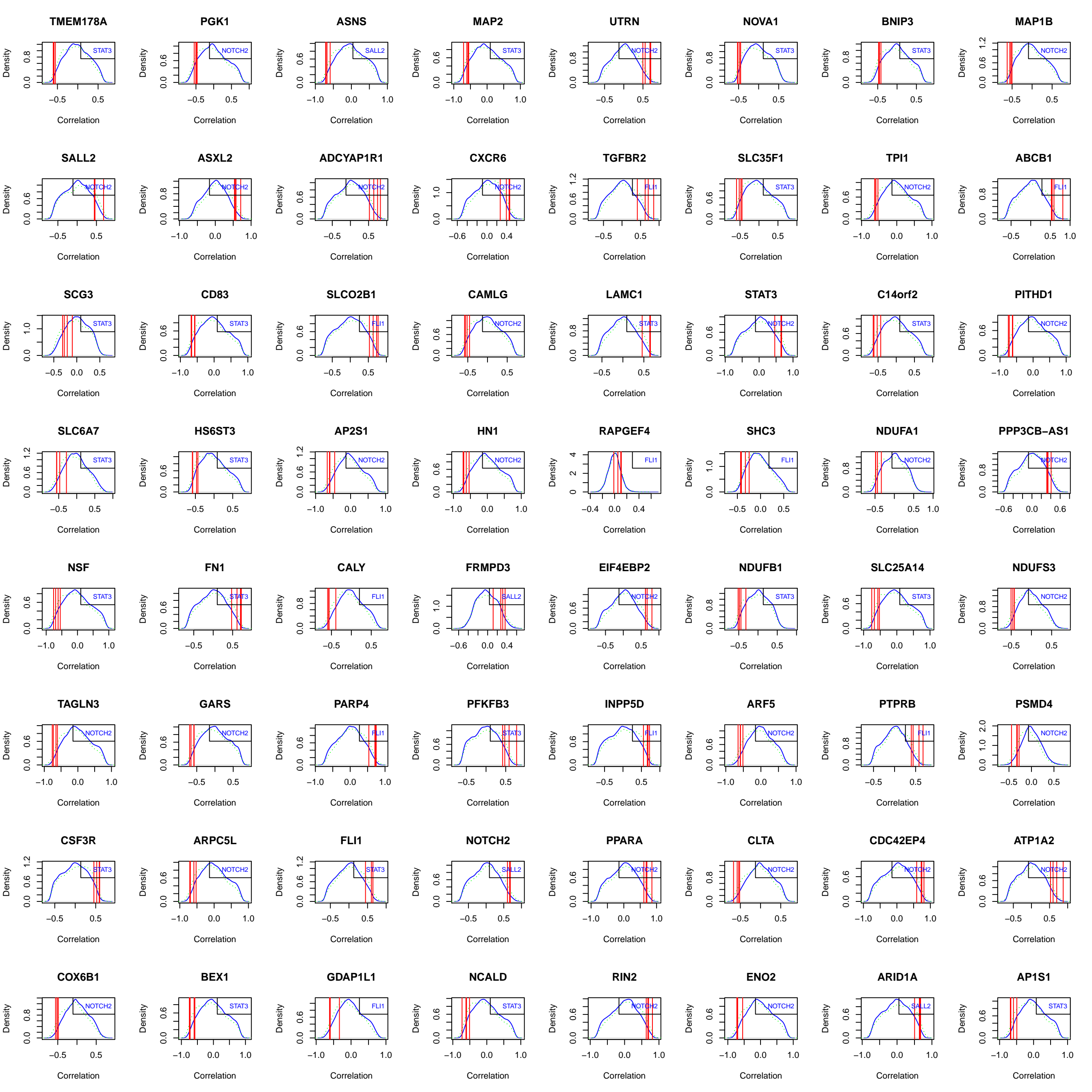

Supplement: Figure S4 — The co-expression between four TF genes and dysregulated genes. The solid lines indicate the co-expression correlation distribution between dysregulated gene and 2045 TF genes; the dashed line indicates the co-expression correlation between dysregulated genes and the genomic genes; the legend shows the TF genes with maximum co-expression correlation with dysregulated genes. [file Data_Sheet_4.PDF]

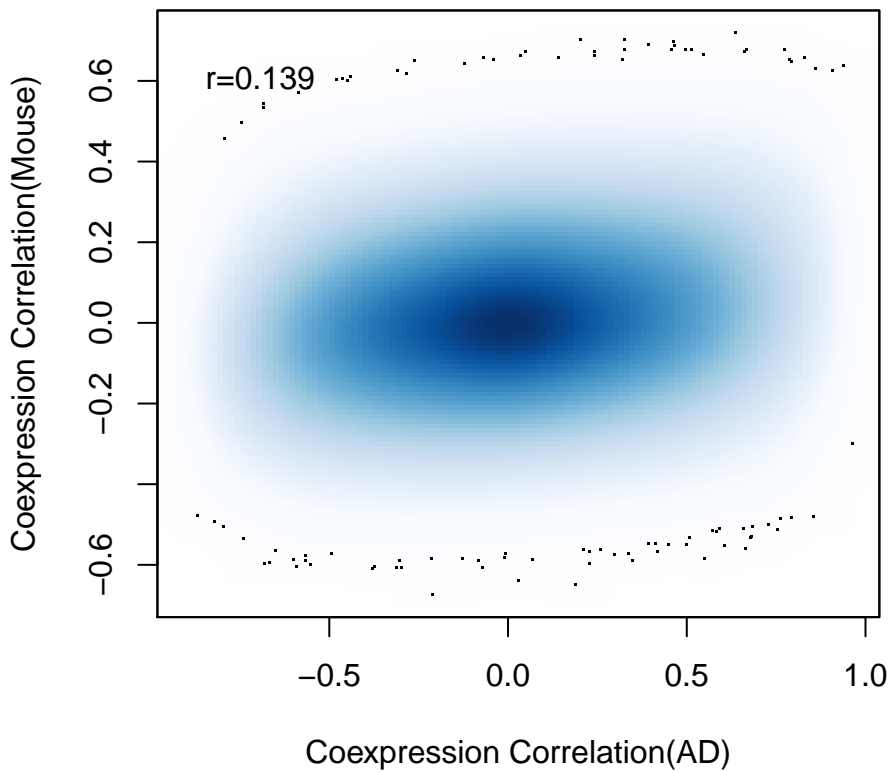

Supplement: Figure S5 — Mouse may have divergent co-expression profile with human AD samples. [file Data_Sheet_5.PDF]

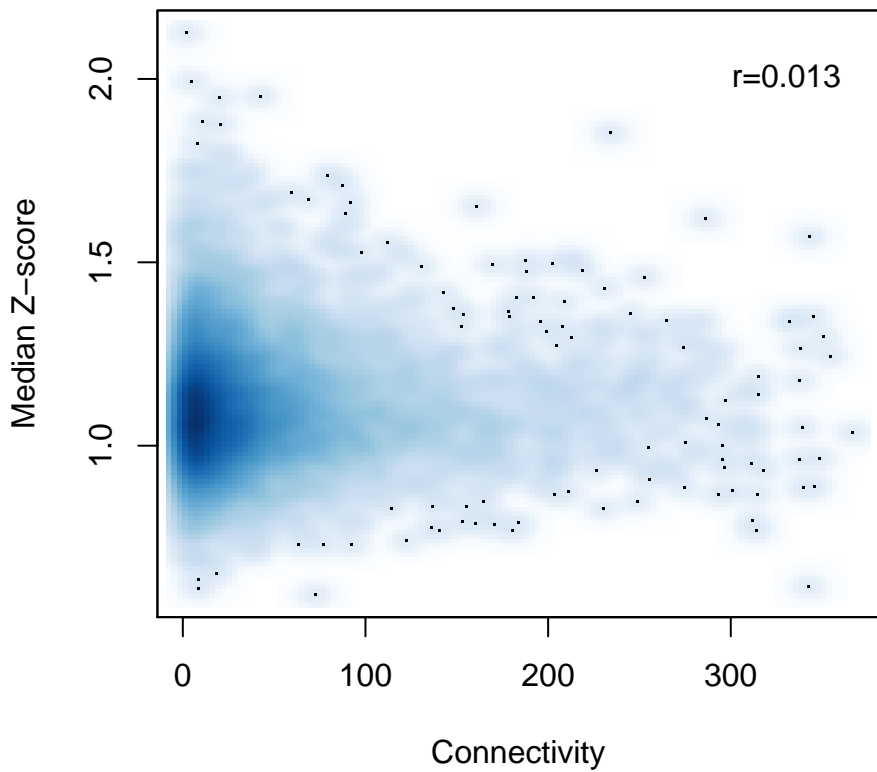

Supplement: Figure S6 — The association between dysregulation and connectivity in the co-expression network. The y-axis shows the median z-score of the differential co-expression. [file Data_Sheet_6.PDF]

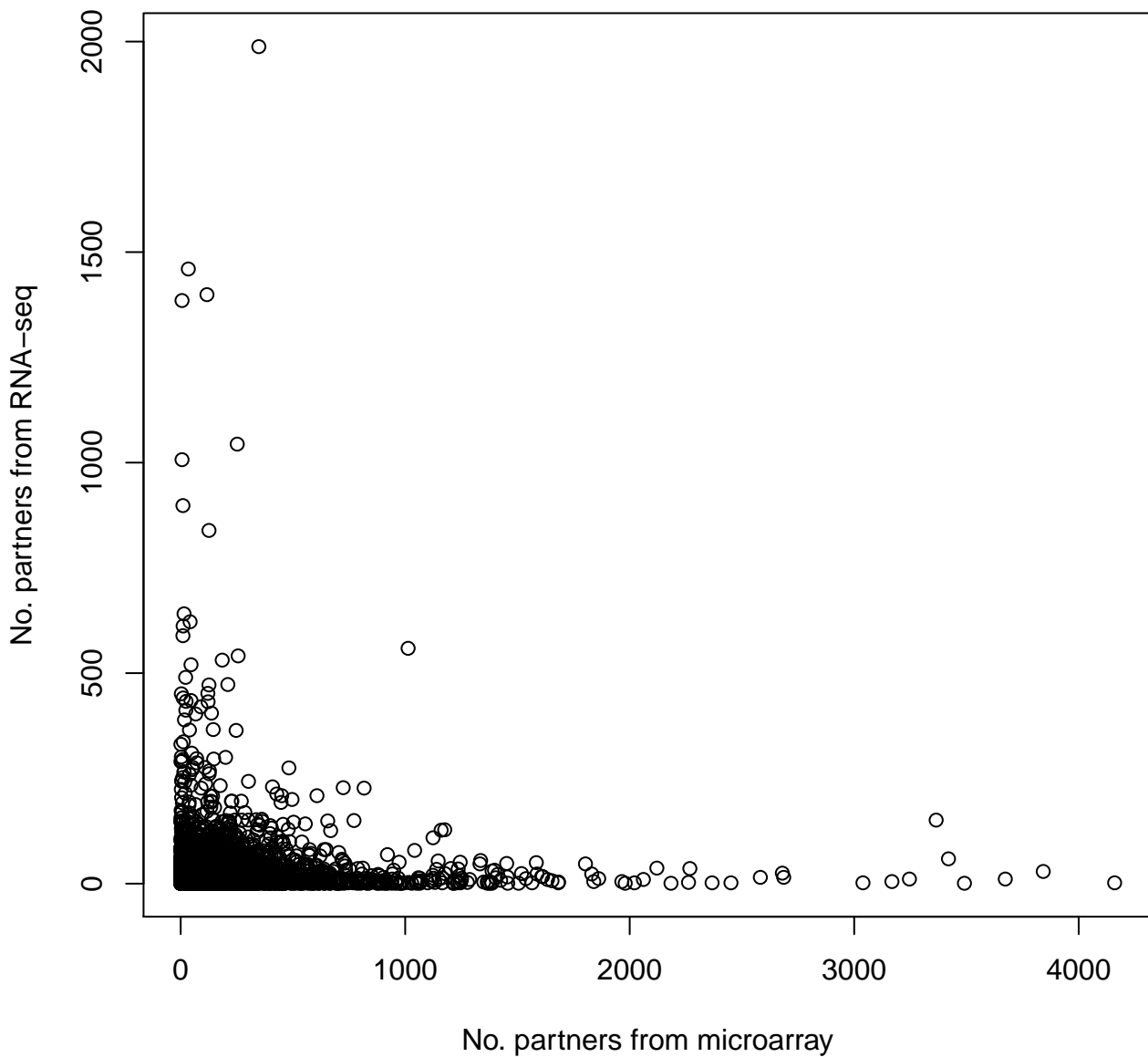

Supplement: Figure S7 — The partner number of dysregulated genes predicted using RNA-seq and microarray data. [file Data_Sheet_7.PDF]

**r= 0.763657609638914**

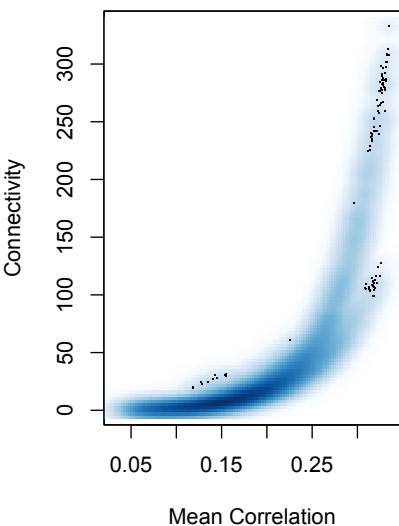

**r= 0.619735354370408**

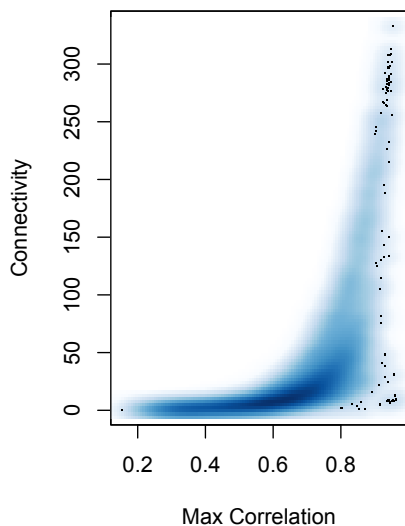

(a)

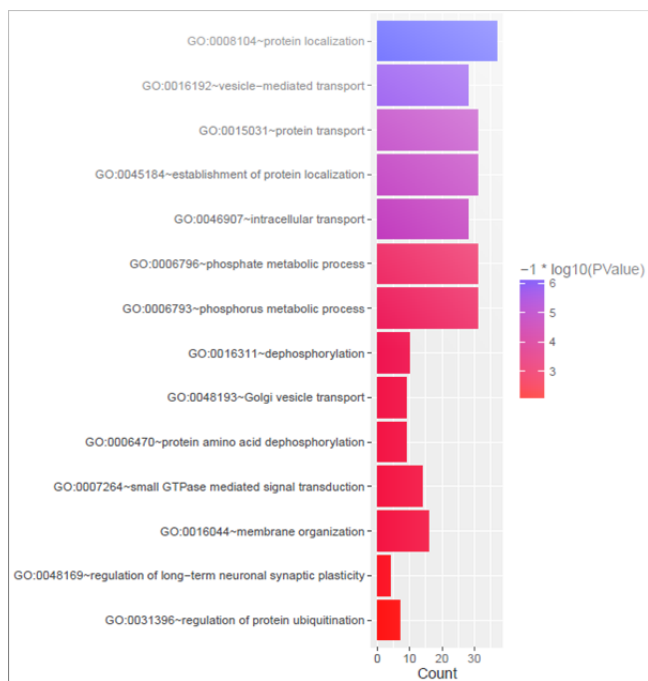

(b)

Supplement: Figure S8 — The association between connectivity and co-expression correlation. (a) the genes with higher connectivity are always the genes with higher co-expression correlation. (b) Functional annotation to the top 200 genes with the highest connectivity. [file Data_Sheet_8.PDF]
