# Supplementary material for: Transcriptional Dysregulation Study Reveals a Core Network Involving the Progression of Alzheimer's Disease
Source: Front Aging Neurosci. 2019 May 7;11:101. doi: 10.3389/fnagi.2019.00101 (PMC6513962; doi:10.3389/fnagi.2019.00101)
Supplement: Doc S1 — The full acknowledgement to the individual data contributors. [file Presentation_1.pdf]

1 Acknowledgment to the Data Contributors

1.1 AMP-AD Consortium

The results published here are in whole or in part based on data obtained from the Accelerating Medicines Partnership for Alzheimer's Disease (AMP-AD) Target Discovery Consortium data portal and can be accessed at doi:10.7303/syn2580853

1.2 ROSMAP RNA-seq data

Study data were provided by the Rush Alzheimers Disease Center, Rush University Medical Center, Chicago. Data collection was supported through funding by NIA grants P30AG10161, R01AG15819, R01AG17917, R01AG30146, R01AG36836, U01AG32984, U01AG46152, the Illinois Department of Public Health, and the Translational Genomics Research Institute.

We thank the patients and their families for their selfless donation to further understanding Alzheimers disease. This project was supported by funding from the National Institute on Aging (AG034504 and AG041232). Many data and biomaterials were collected from several National Institute on Aging (NIA) and National Alzheimers Coordinating Center (NACC, grant #U01 AG016976) funded sites. Amanda J. Myers, PhD (University of Miami, Department of Psychiatry) prepared the series. The directors, pathologist and technicians involved include: Rush University Medical Center, Rush Alzheimer's Disease Center (NIH #AG10161): David A. Bennett, M.D. Julie A. Schneider, MD, MS, Karen Skish, MS, PA (ASCP)MT, Wayne T Longman. The Rush portion of this study was supported by National Institutes of Health grants P30AG10161, R01AG15819, R01AG17917, R01AG36042, R01AG36836, U01AG46152, R01AG34374, R01NS78009, U18NS82140, R01AG42210, R01AG39478, and the Illinois Department of Public Health. Quality control checks and preparation of the gene expression data was provided by the National Institute on Aging Alzheimers Disease Data Storage Site (NIAGADS, U24AG041689) at the University of Pennsylvania.

1.3 Mayo brain RNA-seq data

Study data were provided by the following sources: The Mayo Clinic Alzheimers Disease Genetic Studies, led by Dr. Nilufer Taner and Dr. Steven G. Younkin, Mayo Clinic, Jacksonville, FL using samples from the Mayo Clinic Study of Aging, the Mayo Clinic Alzheimers Disease Research Center, and the Mayo Clinic Brain Bank. Data collection was supported through funding by NIA grants P50 AG016574, R01 AG032990, U01 AG046139, R01 AG018023, U01 AG006576, U01 AG006786, R01 AG025711, R01 AG017216, R01 AG003949, NINDS grant R01 NS080820, CurePSP Foundation, and support from Mayo Foundation. Study data includes samples collected through the Sun Health Research Institute Brain and Body Donation Program of Sun City, Arizona. The Brain and Body Donation Program is supported by the National Institute of Neurological Disorders and Stroke (U24 NS072026 National Brain and Tissue Resource for Parkinsons Disease and Related Disorders), the National Institute on Aging (P30 AG19610 Arizona Alzheimers Disease Core Center), the Arizona Department of Health Services (contract 211002, Arizona Alzheimers Research Center), the Arizona Biomedical Research Commission (contracts 4001, 0011, 05-901 and 1001 to the Arizona Parkinson's Disease Consortium) and the Michael J. Fox Foundation for Parkinsons Research.

1.4 MSBB RNA-seq data

These data were generated from postmortem brain tissue collected through the Mount Sinai VA Medical Center Brain Bank and were provided by Dr. Eric Schadt from Mount Sinai School of Medicine.

1.5 Harvard Brain Bank microarray data

These data were generated from postmortem brain tissue collected through the Harvard Brain Tissue Resource Center and were provided by Dr. Eric Schadt from Mount Sinai School of Medicine.
